# A supervised machine learning approach identifies gene‐regulating factor‐mediated competing endogenous RNA networks in hormone‐dependent cancers

**DOI:** 10.1002/jcb.30300

**Published:** 2022-06-27

**Authors:** Dulari K. Jayarathna, Miguel E. Rentería, Jyotsna Batra, Neha S. Gandhi

**Affiliations:** ^1^ Centre for Genomics and Personalized Health, School of Chemistry and Physics Queensland University of Technology Brisbane QLD Australia; ^2^ Department of Genetics and Computational Biology QIMR Berghofer Medical Research Institute Brisbane QLD Australia; ^3^ School of Biomedical Sciences, Faculty of Health Queensland University of Technology Brisbane QLD Australia; ^4^ Australian Prostate Cancer Research Centre‐Queensland Woolloongabba QLD Australia; ^5^ Cancer and Ageing Research Program Translational Research Institute Woolloongabba QLD Australia

**Keywords:** competing endogenous RNA, copy number alteration, DNA methylation, LASSO regression, machine learning, sparse correlation, transcription factors

## Abstract

Competing endogenous RNAs (ceRNAs) have become an emerging topic in cancer research due to their role in gene regulatory networks. To date, traditional ceRNA bioinformatic studies have investigated microRNAs as the only factor regulating gene expression. Growing evidence suggests that genomic (e.g., copy number alteration [CNA]), transcriptomic (e.g., transcription factors [TFs]), and epigenomic (e.g., DNA methylation [DM]) factors can influence ceRNA regulatory networks. Herein, we used the Least absolute shrinkage and selection operator regression, a machine learning approach, to integrate DM, CNA, and TFs data with RNA expression to infer ceRNA networks in cancer risk. The gene‐regulating factors‐mediated ceRNA networks were identified in four hormone‐dependent (HD) cancer types: prostate, breast, colorectal, and endometrial. The shared ceRNAs across HD cancer types were further investigated using survival analysis, functional enrichment analysis, and protein–protein interaction network analysis. We found two (*BUB1* and *EXO1*) and one (*RRM2*) survival‐significant ceRNA(s) shared across breast‐colorectal‐endometrial and prostate–colorectal–endometrial combinations, respectively. Both *BUB1* and *BUB1B* genes were identified as shared ceRNAs across more than two HD cancers of interest. These genes play a critical role in cell division, spindle‐assembly checkpoint signalling, and correct chromosome alignment. Furthermore, shared ceRNAs across multiple HD cancers have been involved in essential cancer pathways such as cell cycle, p53 signalling, and chromosome segregation. Identifying ceRNAs' roles across multiple related cancers will improve our understanding of their shared disease biology. Moreover, it contributes to the knowledge of RNA‐mediated cancer pathogenesis.

## 1 INTRODUCTION

Historically, the role of protein‐coding genes has been studied extensively in the development and occurrence of cancer. However, in recent years, the investigation of noncoding RNAs (ncRNAs) has increased attention. For example, long noncoding RNAs (lncRNAs) and microRNAs (miRNAs) are two types of ncRNAs that play crucial roles in transcriptional and posttranscriptional gene regulation.^1^ miRNAs are one of the most‐studied regulating factors on gene networks, and their aberrant expression link with the development of several diseases, including cancer.^2^, ^3^, ^4^ The competing endogenous RNA (ceRNA) hypothesis postulates that messenger RNAs (mRNAs) and other RNA transcripts, such as lncRNAs and pseudogenes, can act as natural miRNA sponges.^5^ These RNAs influence each other's expression levels by competing for the same pool of miRNAs through miRNA response elements (MREs) on their target transcripts, thereby modulating gene expression and protein activity.^6^ Crosstalk between ceRNAs through shared MREs represents a novel layer of gene regulation that plays an important role in the physiology and development of complex human diseases such as cancers.

Existing ceRNA network analyses have described many risk‐associated ceRNAs in common cancers.^7^, ^8^, ^9^, ^10^, ^11^ Nevertheless, these bioinformatic analyses have considered miRNAs the only factor regulating gene expression. Additional gene‐regulating factors, namely DNA methylation (DM), transcription factors (TFs), and copy number alteration (CNA), also affect ceRNA regulatory networks.^12^ DM plays a critical role in regulating gene expression through epigenetic control of various biological processes and diseases, including cancer. TFs are commonly deregulated in the pathogenesis of human cancer and are a significant class of cancer cell dependencies.^13^ The CNAs are somatic changes to chromosome structure that results in gain or loss in copies of sections of DNA and are prevalent in many types of cancers. Therefore, current ceRNA‐based computational analyses should be accountable for these genetic and epigenetic effects.^12^

Herein, we used a supervised machine learning approach, ‘Cancerin (Cancer‐associated ceRNA interaction networks)’,^12^ to identify regulation factors‐mediated ceRNA networks in a group of genetically related hormone‐dependent (HD) cancers, using genetic data derived from The Cancer Genome Atlas (TCGA). Cancerin follows three main steps to infer genome‐wide ceRNAs in cancer risk as follows: (i) retrieving putative miRNA–mRNAs and miRNA–lncRNA interactions; (ii) selecting miRNAs that contribute to the RNA expression level; and (iii) inferring ceRNA associations through the hypergeometric test, Pearson correlation analysis and the sparse partial correlation analysis. We identified common ceRNA pairs (mRNA–mRNA or lncRNA–mRNA pairs that have shared the same miRNA[s]) across multiple HD cancers. All cross‐cancer ceRNA pairs obtained from the analysis were involved in survival analysis, functional enrichment analysis, and protein–protein interaction (PPI) network analysis. Identification of the function and mechanism of ceRNAs improves our understanding of RNA‐mediated cancer pathogenesis. Moreover, finding common ceRNAs across HD cancers will significantly contribute to our understanding of the shared biology of HD cancers.

## 2 MATERIALS AND METHODS

### 2.1 Data sets

The genomic and clinical data of five HD cancer types (reposited in TCGA were extracted using the Genomic Data Commons data portal (https://gdc.cancer.gov/access-data/gdc-data-portal).^14^ Cancer types considered in this study were breast invasive carcinoma (BRCA), prostate adenocarcinoma (PRAD), colon adenocarcinoma (COAD), rectum adenocarcinoma (READ) and uterine corpus endometrial carcinoma (UCEC). The PRAD, BRCA, COAD, READ and UCEC consist of 499/52 (cases/controls), 1109/113, 480/41, 167/10 and 552/35, respectively. We retrieved HTSeq‐counts and isoform quantification for RNA and miRNA expression data. Putative miRNA–mRNA and miRNA–lncRNA interactions were downloaded from the starBase (https://starbase.sysu.edu.cn/) and miRcode (http://www.mircode.org/) databases.^15^, ^16^ The miRcode^15^ database facilitates mRNA–miRNA and lncRNA–miRNA target predictions using a broad searchable map that contains 10,419 lncRNAs. The starBase^16^ includes miRNA–mRNA interactions predicted by probing 108 CLIP‐seq data sets. The TF–gene associations were extracted from the Dorothea R package,^17^ covering 1395 TFs and 20,244 genes with 486,676 interactions.

### 2.2 Differential expression analysis

First, samples with duplicated IDs were removed from the retrieved TCGA data set. Then, metastatic samples were excluded as solid tumours and adjacent normal groups were compared in the differential expression analysis. The low‐expressed genes (log counts per million < 1 in more than 50% of the samples) were excluded to increase the sensitivity and precision of the differential expression analysis. After excluding low‐expressed genes, RNA and miRNA raw counts data were re‐normalized using the trimmed mean of M values method implemented in the edgeR R package.^18^ Then data were standardized using the voom method in the linear modelling for microarrays (limma) R package.^19^ Differentially expressed (DE) mRNAs, lncRNAs and miRNAs were identified using a linear modelling method (lmFit function) followed by empirical Bayes (eBayes) moderation. The eBayes method borrows information across all the genes to obtain more precise estimates of gene‐wise variability. According to linear modelling results, |logFC| > 1 and false discovery rate < 0.01 were considered thresholds to identify statistically significant mRNAs, lncRNAs and miRNAs.^20^ The RNA and miRNA expression levels of DE mRNAs, lncRNAs and miRNAs were selected as independent (X)/dependent (Y) variables for machine learning models.

### 2.3 CNA and DM

We obtained gene‐level copy number values and gene‐level DM values on CpG sites (i.e., *β* values) using the Linked Omics database (http://www.linkedomics.org/login.php),^21^ a publicly available resource for multi‐omics data from all 32 cancer types in TCGA. The *β* value was estimated as the ratio of the methylated probe intensity to the overall intensity (sum of methylated and unmethylated intensities). Therefore, the *β* value ranges between 0 and 1, with 0 being hypomethylated and 1 being hypermethylated. Linked Omics^21^ combines COAD and READ copy number values and methylation *β* values. Therefore, we integrated COAD and READ RNA and miRNA expression samples into a single analysis of colorectal cancer (COLCA). We use the ‘COLCA’ term to explain results in the combined analysis of COAD and READ data.

### 2.4 Cancerin: a supervised machine learning approach to identify ceRNA networks in cancer risk

We used a supervised machine learning approach, Cancerin,^12^ to identify genome‐wide ceRNAs in cancer risk. The Cancerin pipeline requires DE RNA expression data, gene‐level copy number values, and gene‐level methylation *β* values from tumour samples. The Cancerin pipeline consists of three main steps as follows:

1. Retrieving putative miRNA–mRNAs and miRNA–lncRNA interactions.
  The putative interactions between DE miRNA and DE mRNA (obtained from the differential expression analysis step) were retrieved from the starBase^16^ and miRcode^15^ databases, where DE miRNA and DE lncRNA associations were extracted from the miRcode.^15^
2. Selecting miRNAs that contribute to the RNA expression level.
  We used a regularized regression method, the least absolute shrinkage and selection operator (LASSO), to evaluate the effect of miRNA regulators on RNA expression levels.^12^ LASSO regression is a type of linear regression that uses shrinkage where data values are shrunk towards a central point such as the mean. The mathematical equation of LASSO regression is given in Equation 1.
(1) $$\sum_{i=1}^{n}{\left(y_{i}-\sum_{j}x_{\mathrm{ij}}\beta_{j}\right)}^{2}+\lambda \sum_{j=1}^{p}\left|\beta_{j}\right|$$
  The blue‐coloured part implies the residual sum of squares calculation, *λ* denotes the amount of shrinkage and the green‐coloured section defines the sum of the absolute value of the magnitude of coefficients. The LASSO models are equivalent to the linear regression models when *λ* = 0. Increasing/decreasing *λ* value cause increased biasedness/increased variance, respectively. We have used the 10‐fold cross‐validation approach to find the optimal *λ* value for each LASSO model.
  The LASSO models are constructed considering transcription‐mediating factors such as DM, TF and CNA. Two model types are fitted for DE mRNAs (see Equation 2) and DE lncRNAs (see Equation 3).
(2) mRNAexpression~CNA+DM+miRNAexpression+TFexpression
(3) lncRNAexpression~miRNAexpression
  We executed every LASSO model 100 times and non‐zero coefficients more than 75 times were selected as LASSO predictors. Unlike multiple linear regression models, LASSO‐selected independent variables are not associated with any statistical significance test. Therefore, we followed the bootstrap procedure for constructing confidence intervals for frequently selected predictors. The subsequent analysis selected the statistically significant mRNA–miRNA and lncRNA–miRNA pairs from LASSO models.
3. Inferring ceRNA associations through the hypergeometric test, Pearson correlation analysis, and the sparse partial correlation analysis.

The Cancerin pipeline introduces three steps to infer ceRNA associations as follows: (i) Pearson correlation analysis to filter strongly positive correlated ceRNA candidate pairs; (ii) hypergeometric test to identify lncRNA–mRNA sharing the significant number of miRNAs; (iii) using the bnlearn R package^22^ to calculate sensitivity correlation (scor) for each candidate ceRNA pair. The scor value in Step iii does not account for a combinatorial effect of multiple miRNAs. Subsequently, strong ceRNAs mediated by multiple moderate miRNA regulators cannot be detected. Therefore, we utilized an extension of scor, the multiple scor (mscor) method, which has been implemented in the Sparse Partial correlation ON Gene Expression R/Bioconductor package.^23^ We used three thresholds as follows: the hypergeometric test *p* < 0.05, the Pearson correlation coefficient of lncRNA–mRNA or mRNA–mRNA pairs > 0.40, and the adjusted *p* of mscor < 0.05 to infer ceRNA candidates of each HD cancer. We have extensively described these methods in our previous conventional ceRNA network analyses paper.^6^

Identifying ceRNAs common among multiple HD cancer types may assist in understanding their shared molecular pathogenesis and drug repositioning. Therefore, we evaluated shared ceRNA pairs (lncRNA–mRNA or mRNA–mRNA) among HD cancer types. The statistically significant ceRNA pairs (RNA A–RNA B) in each HD cancer were compiled into a single table. Then, we coded a new variable combining ceRNA candidate pairs, such as ‘RNA A ensemble ID_RNA B ensemble ID.’ Then, the one‐way table was created to identify shared values of the derived variable across all four HD cancer types. Suppose one‐way frequency equals 4 for a given RNA A–RNA B combination. In that case, a ceRNA association is classified into ‘the shared ceRNA network of HD cancers.’ Similarly, if frequency equals 2 or 3, these ceRNAs have been associated with two or three cancer types, respectively.

Shared ceRNAs across multiple HD cancer types were applied in survival analysis, functional enrichment analysis, and PPI network analysis to evaluate their predictive ability and biological functions in cancer. We used survival^24^ and clusterprofiler^25^ R packages for survival and functional enrichment analyses. The shared ceRNAs among HD cancer combinations were checked using the Kaplan–Meir (K‐M) survival curves in survival analysis. We studied whether a given ceRNA exhibits prognosis ability in individual cancers included in combinations. For example, assume that Gene A is a common ceRNA for BRCA and PRAD, then we check whether Gene A is statistically significant from both BRCA and PRAD survival analyses. The PPI network analyses were conducted using the STRING version 11.5.^26^

## 3 RESULTS

After the quality‐control process, we retrieved 495/52 (cases/controls), 1091/113, 456/41, 166/10, and 543/35 samples from PRAD, BRCA, COAD, READ, and UCEC. In the differential expression analysis, we used 15,509, 15,244, 14,771, 14,866, and 15,197 genes from PRAD, BRCA, COAD, READ, and UCEC after removing those that were low‐expressed.

### 3.1 Differential expression analysis results

The number of DE RNAs, DE miRNAs, DM, CNA, TF–target interactions and miRNA–lncRNA/mRNA interactions used in each HD cancer‐specific ceRNA analysis are given in Table 1.

**Table 1:** The count of DE RNAs and other regulator factors involved in the machine learning‐based ceRNA network analysis

Cancer		RNA	miRNA	DM	CNA	TFs–target interactions	miRNA–lncRNA/mRNA interactions
PRAD		1701	61	19,708	24,079	454,505	7,963,270
BRCA		2934	158	20,106	24,776	454,505	16,608,714
UCEC		3837	245	20,118	24,776	454,505	27,124,806
COLCA							
COAD		3187	339	20,113	24,776	454,505	17,449,352
READ		3193	279	20,113	24,776	454,505	18,422,800

Abbreviations: BRCA, breast invasive carcinoma; ceRNA, competing endogenous RNA; CNA, copy number alteration; COAD, colon adenocarcinoma; COLCA, colorectal cancer; DE, differentially expressed; DM, DNA methylation; lncRNA, long noncoding RNA; miRNA, microRNA; mRNA, messenger RNA; PRAD, prostate adenocarcinoma; READ, rectum adenocarcinoma; TFs, transcription factors; UCEC, uterine corpus endometrial carcinoma.

In the next analytical steps (machine‐learning approach and inferring ceRNAs), DE mRNA/miRNA expression data of COAD and READ combined as colorectal cancer (COLCA) analysis.

### 3.2 Machine‐learning‐based ceRNA network analyses results

In LASSO models, we used DE RNA expression data, DM *β* estimates, CNA values, miRNA–lncRNA/mRNA and TF–target interactions. The statistically significant LASSO predictors (estimated by the bootstrap procedure) were used to identify potential ceRNA pairs. After that, we applied three user‐defined thresholds (see Section 2 for more details) to infer ceRNA interactions. Table 2 describes the number of significant ceRNA associations found in each HD cancer and shared among more than two cancer types. Please see the Supporting Information file for ceRNAs in individual/shared HD cancer(s).

**Table 2:** The number of ceRNAs in individual HD cancer networks/shared among cancer combinations

Cancer or cancer combinations	Count of statistically significant ceRNA combinations (number of unique ceRNAs)
PRAD	1802 (280)
BRCA	9340 (963)
COLCA (COAD ∪READ)	2858 (750)
UCEC	3165 (604)
PRAD ∩BRCA	37 (35)
PRAD ∩COLCA	5 (9)
PRAD ∩UCEC	15 (16)
BRCA ∩COLCA	70 (72)
BRCA ∩UCEC	202 (88)
COLCA ∩UCEC	77 (60)
PRAD ∩BRCA ∩COLCA	0
PRAD ∩BRCA ∩UCEC	2 (4)
BRCA ∩COLCA ∩UCEC	9 (16)
PRAD ∩COLCA ∩UCEC	2 (3)
PRAD ∩BRCA ∩ COLCA ∩UCEC	0

*Note*: ∩ denotes the intersection between given data sets. For instance, PRAD∩BRCA implies the set of shared competing endogenous RNAs among prostate and breast cancers.

Abbreviations: BRCA, breast invasive carcinoma; ceRNA, competing endogenous RNA; COAD, colon adenocarcinoma; COLCA, colorectal cancer; HD, hormone‐dependent; PRAD, prostate adenocarcinoma; READ, rectum adenocarcinoma; UCEC, uterine corpus endometrial carcinoma.

As shown in Table 2, most ceRNAs were unique for individual cancer types. BRCA and UCEC have shared the highest ceRNA candidates compared with other cancers. All significant ceRNAs have been derived from mRNA–mRNA pairs and none of the lncRNA–mRNA pairs was observed across multiple HD cancers.

### 3.3 Survival analysis

The ceRNAs common among HD cancer combinations were checked for the prognosis in each cancer (included in the combination). Table 3 describes significant ceRNAs from each HD cancer combination with their hazard ratios and *p* values. The hazard ratio is defined as the slope of the survival curve, which measures how rapidly the event (death) has occurred. The hazard ratio compares two groups, here low and high expressed. The hazard ratios and *p* values in blue/red fonts indicate that the gene's low/high expressed level is associated with the survival of cancer in interest. In Table 3, the significant ceRNAs from COAD‐READ combined analysis (COLCA) were checked for prognostic in COAD/READ.

**Table 3:** Survival significant ceRNAs in the combinations of HD cancers

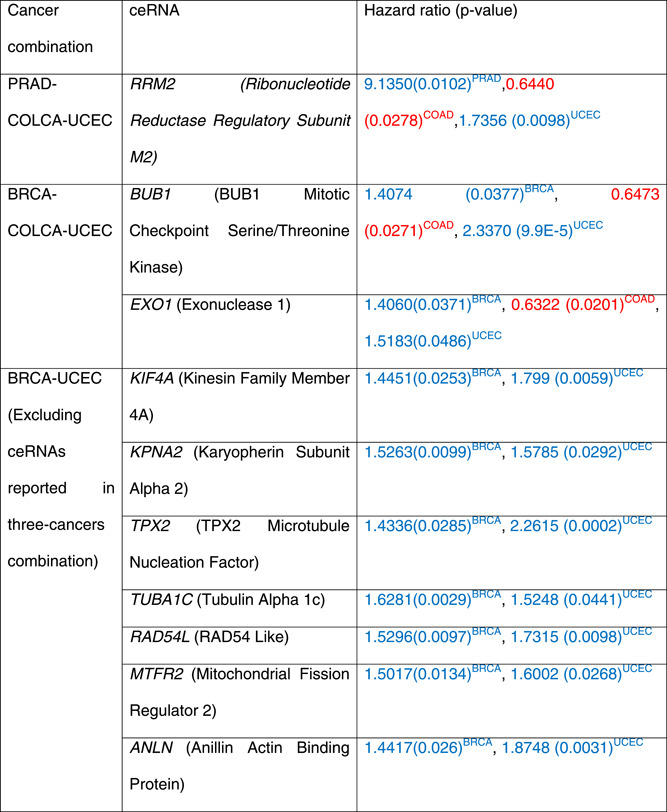
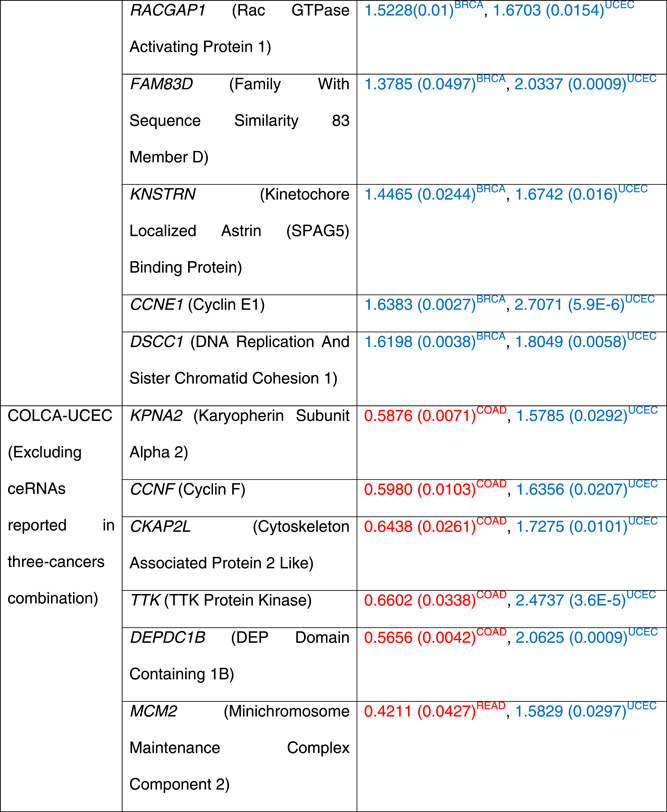

*Note*: The hazard ratios and *p* in blue/red fonts indicate that the gene's low/high expressed level is associated with the survival of cancer in interest.

Abbreviations: BRCA, breast invasive carcinoma; ceRNA, competing endogenous RNA; COAD, colon adenocarcinoma; COLCA, colorectal cancer; HD, hormone‐dependent; PRAD, prostate adenocarcinoma; READ, rectum adenocarcinoma; UCEC, uterine corpus endometrial carcinoma.

As described in Table 3, most survival‐significant ceRNAs have been shared among BRCA and UCEC. Previous studies have also shown the shared genetic susceptibility of BRCA and UCEC.^14^, ^27^ Two ceRNAs (*BUB1* and *EXO1*) from the BRCA‐COAD‐UCEC and the *RRM2* from PRAD‐COAD‐UCEC were prognostic in all three cancer types included in the combination of interest.

**Figure 1:**
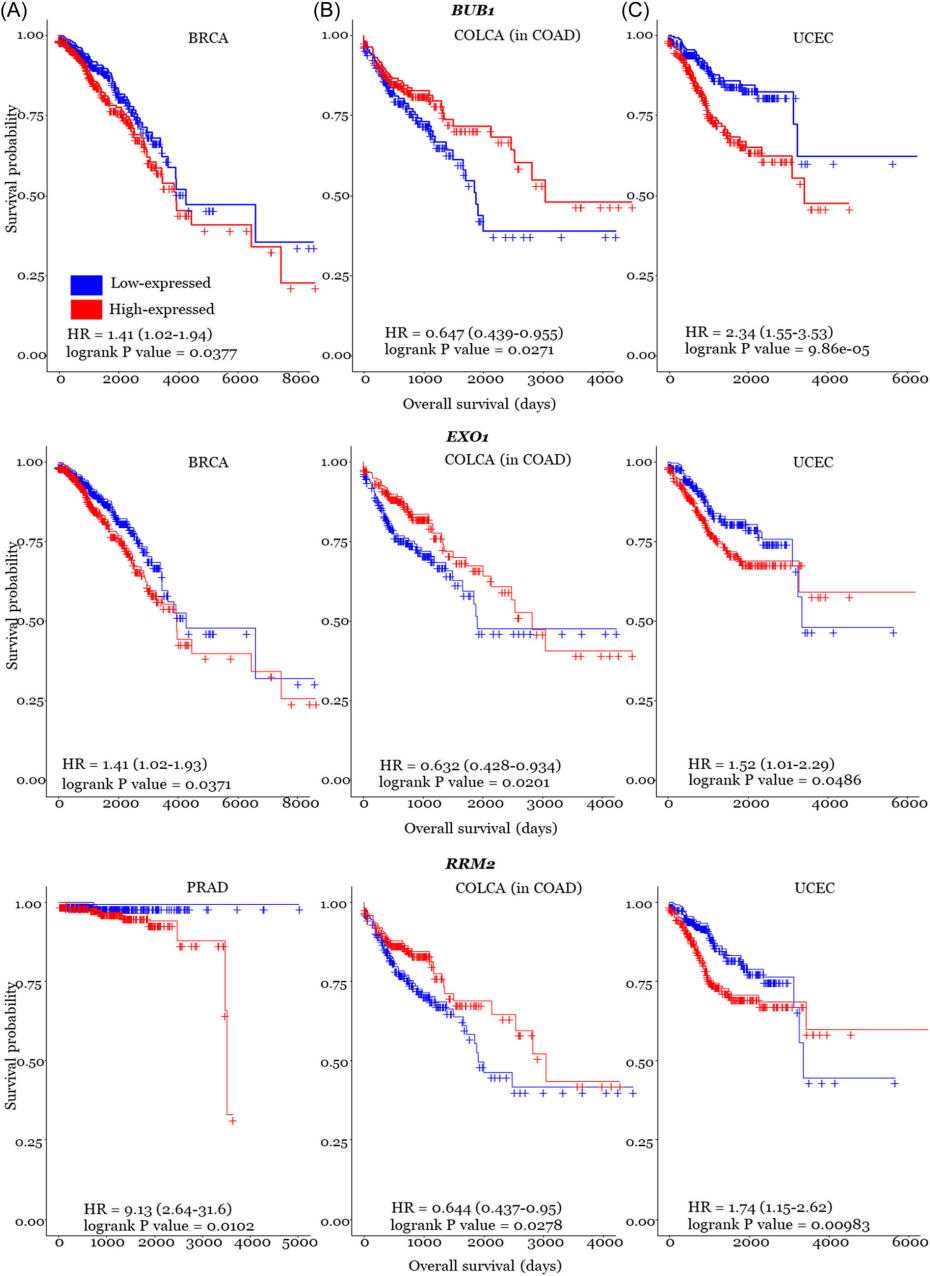
Kaplan–Meier survival curves for the shared competing endogenous RNAs (ceRNAs) across three hormone‐dependent (HD) cancers. The *BUB1* (top) and *EXO1* (middle) genes are prognostic in breast (A), colon (B) and endometrial (C) cancers. The *RRM2* (bottom) is survival significant in prostate (A), colon (B) and endometrial (C) cancers.

The K‐M survival curves for *BUB1* (in BRCA, COAD and UCEC), *EXO1* (BRCA, COAD and UCEC), and *RRM2* (PRAD, COAD and UCEC) are illustrated in Figure 1.

In BRCA (A) and UCEC (C), low‐expressed *BUB1* and *EXO1* show better survival. Nevertheless, high‐expressed *BUB1* and *EXO1* are associated with a higher survival probability in COAD (B). The low‐expressed *RRM2* gene has shown the highest survival probability (hazard ratio = 9.13) in PRAD (A) compared with other cancers, COAD (B) and UCEC (C).

### 3.4 Functional enrichment analysis

We conducted the functional enrichment analysis for shared ceRNAs in each HD cancer combination. The genes shared across two/three HD cancers (listed in Supporting Information) was used for the functional enrichment analysis. Table 4 reports the top‐five components of Gene Ontology (GO) and Kyoto Encyclopedia of Genes and Genomes (KEGG) pathways for each two‐HD cancer combination‐associated ceRNAs. Please see the Supporting Information file for list of genes in each GO/KEGG pathway.

**Table 4:** Functional enrichment analyses result for shared ceRNAs across two HD cancers combinations

Cancer combination	Terms (GO/KEGG pathway)	FDR
PRAD∩BRCA	Mitotic sister chromatid segregation (GO)	1.44E − 19
	Mitotic nuclear division (GO)	1.44E − 19
	Chromosome segregation (GO)	7.54E − 19
	Organelle fission (GO)	8.46E − 18
	Regulation of chromosome segregation (GO)	4.41E − 15
	Cell cycle (KEGG)	2.26E − 05
	Oocyte meiosis (KEGG)	0.008609
PRAD∩COLCA	Condensed chromosome (GO)	0.001566
	Mitotic chromosome condensation (GO)	0.002994
	Mitotic sister chromatid segregation (GO)	0.002994
	Condensed nuclear chromosome (GO)	0.004676
	DNA packaging complex (GO)	0.004676
PRAD∩UCEC	Chromosome segregation (GO)	2.38E − 18
	Sister chromatid segregation (GO)	7.4E − 17
	Nuclear division (GO)	2.19E − 15
	Mitotic nuclear division (GO)	2.21E − 15
	Regulation of chromosome segregation (GO)	4.29E − 13
	Cell cycle (KEGG)	0.042667
BRCA∩COLCA	Nuclear division (GO)	4.61E − 20
	Chromosome segregation (GO)	5.05E − 19
	Mitotic nuclear division (GO)	5.54E − 19
	Mitotic sister chromatid segregation (GO)	3.59E − 16
	Chromosomal region (GO)	3.38E − 12
	Cell cycle (KEGG)	1.97E − 12
	Oocyte meiosis (KEGG)	2.05E − 05
	Progesterone‐mediated oocyte maturation (KEGG)	5.45E − 05
	p53 signalling pathway (KEGG)	0.000205
	Cellular senescence (KEGG)	0.00052
BRCA∩UCEC	Chromosome segregation (GO)	2.41E − 27
	Chromosomal region (GO)	5.22E − 26
	DNA replication (GO)	1.5E − 22
	Organelle fission (GO)	8.05E − 22
	Nuclear division (GO)	8.05E − 22
	Cell cycle (KEGG)	3.06E − 14
	DNA replication (KEGG)	7.94E − 06
	Mismatch repair (KEGG)	0.001704
	p53 signalling pathway (KEGG)	0.002887
	Cellular senescence (KEGG)	0.004181
COLCA∩UCEC	Chromosome segregation (GO)	8.31E − 18
	Chromosomal region (GO)	1.12E − 16
	Mitotic nuclear division (GO)	2.86E − 16
	Nuclear division (GO)	5.24E − 16
	DNA replication (GO)	1.42E − 15
	Cell cycle (KEGG)	2.88E − 10
	DNA replication (KEGG)	0.000148
	p53 signalling pathway (KEGG)	0.001661
	Fanconi anaemia pathway (KEGG)	0.007581
	Oocyte meiosis (KEGG)	0.007581

Abbreviations: BRCA, breast invasive carcinoma; ceRNAs, competing endogenous RNAs; COAD, colon adenocarcinoma; COLCA, colorectal cancer; FDR, false discovery rate; GO, Gene Ontology; HD, hormone‐dependent; KEGG, Kyoto Encyclopedia of Genes and Genomes; PRAD, prostate adenocarcinoma; READ, rectum adenocarcinoma; UCEC, uterine corpus endometrial carcinoma.

**Figure 2:**
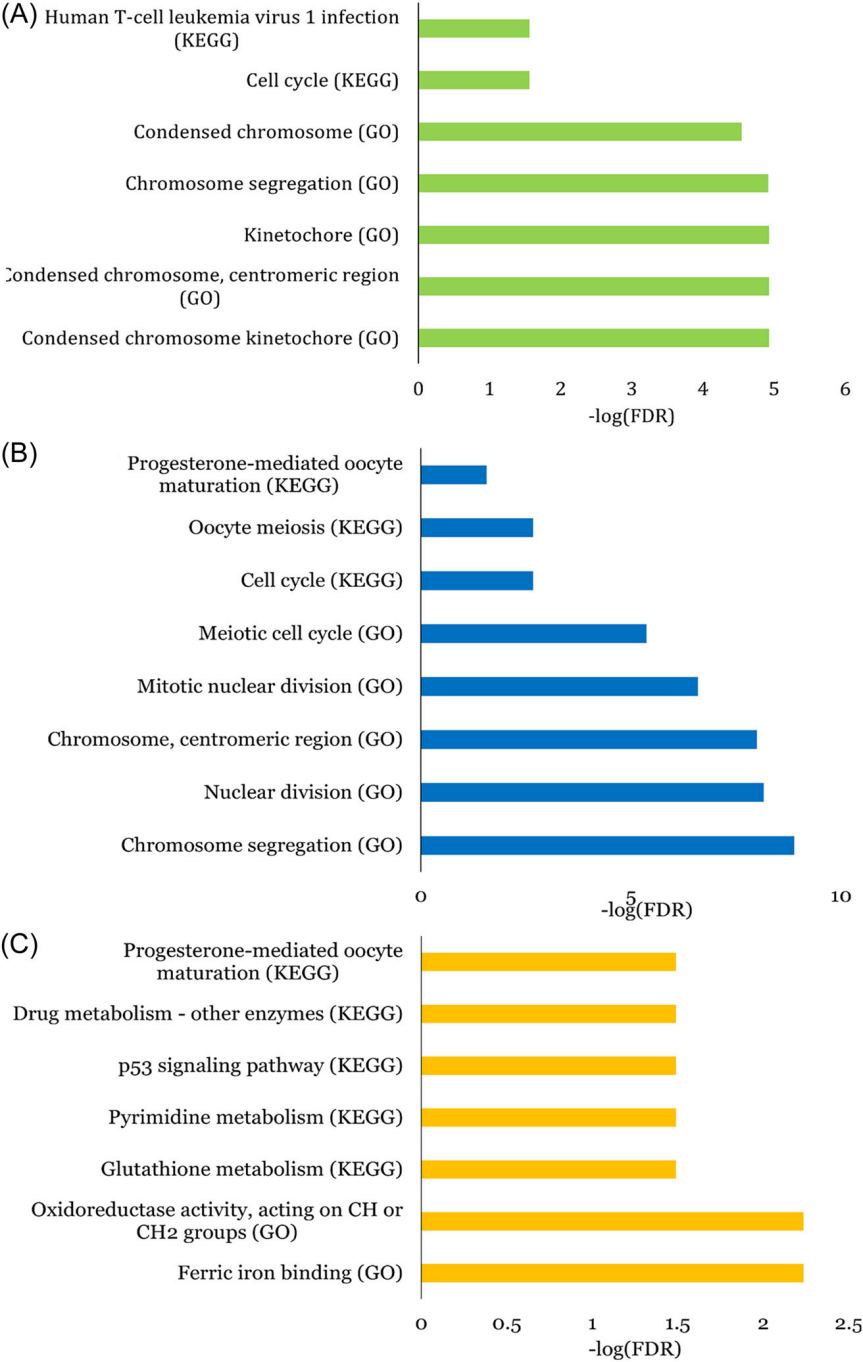
Top‐five Gene Ontology (GO) and Kyoto Encyclopedia of Genes and Genome (KEGG) pathway components for shared ceRNAs across prostate‐breast‐endometrial (A), breast‐colorectal‐endometrial (C), and prostate‐colorectal‐endometrial (C).

Enrichment analyses result of ceRNAs shared among three‐HD cancers are illustrated in Figure 2.

### 3.5 PPI network analysis results

PPI network analyses for ceRNAs shared among HD cancer combinations were conducted using the STRING online platform.^26^ The minimum required interaction score of the PPI networks was adjusted to the highest confidence level (0.900) to improve the strength of the interaction network. Table 5 describes the PPI network analyses results.

**Table 5:** PPI network analysis result for each ceRNAs set shared among HD cancer combinations

Cancer combinations	Number of nodes	Number of edges	PPI enrichment *p*
PRAD∩BRCA	35	141	<1.0E − 16
PRAD∩COLCA	9	7	6.68E − 10
PRAD∩UCEC	16	67	<1.0E − 16
BRCA∩COLCA	72	193	<1.0E − 16
BRCA∩UCEC	88	332	<1.0E − 16
COLCA∩UCEC	60	150	<1.0E − 16
PRAD∩BRCA∩UCEC	4	6	1.12E − 09
BRCA∩COLCA∩UCEC	16	10	2.04E − 09
PRAD∩COLCA∩UCEC	3	1	0.0354

Abbreviations: BRCA, breast invasive carcinoma; ceRNAs, competing endogenous RNAs; COLCA, colorectal cancer; HD, hormone‐dependent; PPI, protein–protein interaction; PRAD, prostate adenocarcinoma; UCEC, uterine corpus endometrial carcinoma.

## 4 DISCUSSION

The aetiology of complex diseases such as cancer is often related to aberrant gene expression at the transcriptional and posttranscriptional levels. Over the last decade, ceRNAs have emerged as an important class of posttranscriptional regulators that alter gene expression through a miRNA‐mediated mechanism. This study has extended the knowledge of ceRNA networks in HD cancers (PRAD, BRCA, COLCA and UCEC) by integrating other molecular effects such as DM, TF and CNA, which influence the ceRNA mechanism. Moreover, it expands the knowledge of HD cancers shared biology by investigating and comparing gene regulatory networks of more than two HD cancer types.

First, we investigated regulating factors‐mediated ceRNAs in individual HD cancers. After that, we identified ceRNAs shared across two/three/four HD cancers. However, none of the ceRNAs was shared among the four cancers of interest. The shared ceRNAs across two/three HD cancers were involved in survival analysis and two downstream analyses, functional enrichment analysis and PPI network analysis.

We found 16 ceRNAs shared across BRCA, COLCA, and UCEC. Among them, *BUB1* and *EXO1* were associated with patient survival of all three cancers of interest. *BUB1* gene encodes a serine/threonine‐protein kinase that plays a central role in mitosis. It also involves DNA damage response and cell cycle regulation.^28^ The *BUB1* gene has been previously reported in BRCA, COLCA, and multiple digestive tract cancers but not in UCEC.^29^ Herein, *BUB1* appears for the first time in UCEC risk. The *BUB1 β* (*BUB1B*) was recognized as a shared ceRNA among PRAD, BRCA and UCEC. These *BUB1* and *BUB1B* genes are central components of the mitotic checkpoint for spindle assembly (SAC).^29^ The SAC plays a fundamental role in maintaining genome stability by ensuring the timely segregation of the genetic material at every cell division. Mistakes in the cell division process can lead to rearrangement, loss or gain of chromosomes, genome instability and cancers.^30^ Therefore, the molecular function of *BUB1* and *BUB1B* genes in HD cancers should be further examined. Several experimental and bioinformatic studies have shown the importance of *EXO1* in replication, DNA repair pathways, cell cycle checkpoints, and its association to cancer.^31^ Genome‐level studies have identified specific mutations in the *EXO1* gene as risk alleles for different types of cancers.^32^, ^33^

Three genes, *DEPDC1B*, *BUB1* and *RRM2*, were identified as shared ceRNAs among PRAD, COLCA and UCEC. *DEPDC1B* has been identified as a prostate cancer metastasis oncogene. It was positively correlated with metastasis status, high Gleason score, advanced tumour stage and poor prognosis. Moreover, mechanistic investigations found that *DEPDC1B* induced epithelial–mesenchymal transition (EMT) and enhanced proliferation by binding to Rac1 and enhancing the Rac1‐PAK1 pathway.^34^ EMT plays an important role in empowering cancer cells to adapt and survive at the start of the metastatic stage. The role of *DEPDC1B* in COLCA and UCEC has not been described in previous bioinformatics or functional studies. According to the survival analysis result, the *RRM2* gene was a prognostic biomarker in PRAD, COLCA and UCEC. The well‐established role of *RRM2 i*s to maintain the balance of deoxynucleoside triphosphate pools for DNA synthesis and DNA repair.^35^ The tumour‐promoting feature of *RRM2* is associated with inducing activities of various oncogenes, including those encoding nuclear factor‐κB, Myc proto‐oncogene protein, tyrosine‐protein kinase transforming protein Fes and ornithine decarboxylase.^36^ The overexpression of *RRM2* correlates with cellular invasiveness, metastasis and tumourigenesis of cervical^37^ and gastric^38^ cancers. Aberrant *RRM2* expression has also been described in ovarian^39^ and prostate^40^ cancers. Liu et al.^41^ have described that *RRM2* serves as a prognostic biomarker in colorectal cancer. None of the ceRNAs was shared among PRAD, BRCA and COLCA, and hence common ceRNAs were not found across four HD cancer types (PRAD, BRCA, COLCA and UCEC) included in this study.

The survival analyses of shared ceRNAs among two HD cancer combinations have shown that 12 (*KIF4A*, *KPNA2*, *TPX2*, *TUBA1C*, *RAD54L*, *MTFR2*, *ANLN*, *RACGAP1*, *FAM83D*, *KNSTRN*, *CCNE1* and *DSCC1*) out 88 and 6 (*KPNA2*, *CCNF*, *CKAP2L*, *TTK*, *DEPDC1B* and *MCM2*) out of 77 ceRNAs shared among BRCA‐UCEC and COLCA‐UCEC pairs, respectively, have exhibited the survival significance in both cancers included in the combinations. These 18 genes have been previously described in HD studies due to their tumour‐suppressive/oncogenic/cancer‐driven nature.^42^

We conducted a functional enrichment analysis for common ceRNAs across two/three HD cancers combinations. The shared ceRNAs across HD cancers have mainly been involved in mitosis (mitotic sister chromatid segregation, mitotic nuclear division and mitotic chromosome condensation) and chromosome related GO pathways (chromosome segregation, regulation of chromosome segregation, condensed chromosome, condensed nuclear chromosome and chromosomal region), which play an important role in cancer. The shared ceRNAs across PRAD, COLCA and UCEC were involved in five unique KEGG pathways, drug metabolism‐other enzymes, pyrimidine metabolism, glutathione metabolism, oxidoreductase activity, acting on CH or CH2 groups, and ferric iron‐binding. Experimental studies might be required to investigate these KEGG pathways' involvement in the shared biology of PRAD, COLCA and UCEC.

The PPI network describes the physical interaction between identified shared ceRNAs in this study, mediating the assembly of proteins into protein complexes, for example, mediating signalling/regulation and transport events in the cell. According to PPI network analysis results, ceRNAs lists of each two/three HD cancer combinations have interacted with stronger statistical evidence. Five out of nine HD cancer combinations' PPI network enrichment *p* was <1.0E − 16. Four and 16 ceRNAs shared among PRAD‐BRCA‐UCEC (*p* = 1.12E − 09) and BRCA‐COLCA‐UCEC (*p* = 2.04E − 09) exhibited strong PPI network connecting all the ceRNA nodes. Therefore, these gene sets should be further studied to evaluate their combined effect on HD cancers.

The major limitation of this study is restricting miRNA–mRNA and miRNA–lncRNA selection (in Step 1). We have chosen both predicted and experimentally validated miRNA–target interaction only from two miRNA–mRNA/lncRNA databases, starBase and miRcode. We followed this limitation to get a substantial set of miRNA–mRNA/lncRNA interactions. Consideration of more possible ceRNA associations will improve the machine‐learning outcome by increasing the number of predictors. Moreover, we have not included circRNAs and pseudogenes in the ceRNA network analyses. This study retrieved only a limited number of statistically significant miRNAs from the differential expression analysis of miRNA‐seq data in TCGA‐PRAD. Therefore, the number of miRNAs (independent variables) involved in PRAD‐LASSO models was comparatively lower. Nevertheless, selections of TFs‐target interactions and CNAs were independent of PRAD differential expression analyses.

Our previous bioinformatic study identified shared ceRNAs (both lncRNAs and mRNAs) across PRAD, BRCA, COLCA and UCEC, considering miRNAs as the only gene regulator in the ceRNA network.^6^ This study has integrated conventional ceRNA networks with DM, TF and CNA data using a machine‐learning approach, ‘Cancerin.’ According to our results, DM, TF and CNA significantly have cut‐off shared ceRNAs across HD cancers found from the conventional methods. According to LASSO models, none of the lncRNAs was identified as a shared ceRNA among two/three HD cancers of interest. These results imply that other regulating factors such as DM, TF and CNA play a vital role in the ceRNA regulatory network apart from miRNAs. Therefore, existing statistical and computational methods should be improved to interpret these gene‐regulating factors‐mediated ceRNAs in diseases of interest.

Our machine learning‐based study of omics data provides a set of pivotal ceRNAs associated with two or three HD cancers of interest. According to functional enrichment analyses, shared ceRNAs across two/three HD cancers are involved in hallmarks of cancer, such as cell cycle, DNA replication/repair, chromosomal segregation, mitotic checkpoint, nuclear division, p53 signalling, glutathione metabolism, pyrimidine metabolism and ferric iron binding. Most of these pathways are directly/indirectly associated with RNA‐processing steps that influence RNA regulatory networks, including ceRNAs. A recent study found the complex biology of glutamine (precursor of glutathione) metabolism in driving BRCA growth.^43^ We found glutathione metabolism as a shared pathway among multiple HD cancers. Therefore, future functional studies are required to investigate the biology of glutathione in HD cancers. Dysfunction of pyrimidine metabolism is closely related to cancer progression. Moreover, various drugs targeting pyrimidine metabolism have been approved for multiple cancer types, including BRCA.^44^ This study has introduced bioinformatics analyses into the mechanistic understanding of HD cancers. A natural progression of this study is to identify the link between shared ceRNAs and respective molecular pathways. Nevertheless, these findings will bring novel insights to explore the underlying shared molecular mechanism of HD cancers.

## 5 CONCLUSIONS

It is noteworthy that, to our knowledge, this is the first study that integrates genomic (CNA), transcriptomic (mRNA, lncRNA, miRNA and TF) and epigenetic (DM) regulatory factors to infer genome‐wide ceRNA interactions shared across a group of cancers. These cross‐HD cancer ceRNAs have shown tumour‐suppressive/tumour‐promoting and survival‐prognosis properties in previous HD cancer studies. The main bottleneck of this machine learning approach is the lack of RNA, DM and CNA data from a single study, especially for other diseases, except for cancers. Besides these limitations, cross‐cancer shared ceRNAs may help identify potential unexpected targets applied for a subset of cancers (e.g., HD). Furthermore, it will help to understand the shared disease biology of HD cancers.

## AUTHOR CONTRIBUTIONS

Dulari K. Jayarathna, Neha S. Gandhi, Miguel E. Rentería and Jyotsna Batra designed the study. Dulari K. Jayarathna performed data acquisition, data analysis and data interpretation. Dulari K. Jayarathna wrote the original manuscript. Neha S. Gandhi, Miguel E. Rentería and Jyotsna Batra revised the manuscript critically. All authors read and approved the final manuscript.

## CONFLICT OF INTEREST

The authors declare no conflict of interest.

## ETHICS STATEMENT

This study was performed in line with the principles of the Declaration of Helsinki. Approval was granted by the Ethics Committees of Queensland University of Technology (19 December 2019/1900001147) and QIMR Berghofer Medical Research Institute (23 August 2019/P1051).

## Supporting information

Supplementary information.

## Data Availability

The author confirms that data from the public database for this study have explained the source of the data in detail in the manuscript and provided a link address.

## References

[1] Bhaskaran M , Mohan M . MicroRNAs: history, biogenesis, and their evolving role in animal development and disease. Vet Pathol. 2014;51:759‐774. 10.1177/0300985813502820 24045890 PMC4013251

[2] Matin F , Jeet V , Clements JA , Yousef GM , Batra J . MicroRNA theranostics in prostate cancer precision medicine. Clin Chem. 2016;62:1318‐1333. 10.1373/clinchem.2015.242800 27540032

[3] Peng Y , Croce CM . The role of microRNAs in human cancer. Signal Transduct Target Ther. 2016;1:15004. 10.1038/sigtrans.2015.4 29263891 PMC5661652

[4] Tüfekci KU , Oner MG , Meuwissen RL , Genç S . The role of microRNAs in human diseases. Methods Mol Biol. 2014;1107:33‐50. 10.1007/978-1-62703-748-8_3 24272430

[5] Salmena L , Poliseno L , Tay Y , Kats L , Pandolfi PP . A ceRNA hypothesis: the Rosetta Stone of a hidden RNA language. Cell. 2011;146:353‐358. 10.1016/j.cell.2011.07.014 21802130 PMC3235919

[6] Jayarathna DK , Rentería ME , Sauret E , Batra J , Gandhi NS . Identifying complex lncRNA/pseudogene–miRNA–mRNA crosstalk in hormone‐dependent cancers. Biology. 2021;10:10. 10.3390/biology10101014 PMC8533463 34681112

[7] Guo L , Yang G , Kang Y , et al. Construction and analysis of a ceRNA network reveals potential prognostic markers in colorectal cancer. Front Genet. 2020;11:418. 10.3389/fgene.2020.00418 32457800 PMC7228005

[8] Ouyang D , Li R , Li Y , et al. Construction of a competitive endogenous RNA network in uterine corpus endometrial carcinoma. Med Sci Monit. 2019;25:7998‐8010. 10.12659/msm.915798 31650984 PMC6825398

[9] Tuersong T , Li L , Abulaiti Z , Feng S . Comprehensive analysis of the aberrantly expressed lncRNA‑associated ceRNA network in breast cancer. Mol Med Rep. 2019;19:4697‐4710. 10.3892/mmr.2019.10165 31059025 PMC6522813

[10] Wang Y , Huang T , Sun X , Wang Y . Identification of a potential prognostic lncRNA‐miRNA‐mRNA signature in endometrial cancer based on the competing endogenous RNA network. J Cell Biochem. 2019;120:18845‐18853. 10.1002/jcb.29200 31338870 PMC6771803

[11] Xu N , Wu YP , Yin HB , Xue XY , Gou X . Molecular network‐based identification of competing endogenous RNAs and mRNA signatures that predict survival in prostate cancer. J Transl Med. 2018;16:274. 10.1186/s12967-018-1637-x 30286759 PMC6172814

[12] Do D , Bozdag S . Cancerin: a computational pipeline to infer cancer‐associated ceRNA interaction networks. PLoS Comput Biol. 2018;14:e1006318. 10.1371/journal.pcbi.1006318 30011266 PMC6072113

[13] Bhagwat AS , Vakoc CR . Targeting transcription factors in cancer. Trends Cancer. 2015;1:53‐65. 10.1016/j.trecan.2015.07.001 26645049 PMC4669894

[14] Kazerouni N , Schairer C , Friedman HB , Lacey JV Jr , Greene MH . Family history of breast cancer as a determinant of the risk of developing endometrial cancer: a nationwide cohort study. J Med Genet. 2002;39:826‐832. 10.1136/jmg.39.11.826 12414823 PMC1735013

[15] Jeggari A , Marks DS , Larsson E . miRcode: a map of putative microRNA target sites in the long non‐coding transcriptome. Bioinformatics. 2012;28:2062‐2063. 10.1093/bioinformatics/bts344 22718787 PMC3400968

[16] Li JH , Liu S , Zhou H , Qu LH , Yang JH . starBase v2.0: decoding miRNA‐ceRNA, miRNA‐ncRNA and protein‐RNA interaction networks from large‐scale CLIP‐Seq data. Nucleic Acids Res. 2014;42(D92‐7):92‐97. 10.1093/nar/gkt1248 PMC3964941 24297251

[17] Garcia‐Alonso L , Holland CH , Ibrahim MM , Turei D , Saez‐Rodriguez J . Benchmark and integration of resources for the estimation of human transcription factor activities. Genome Res. 2019;29:1363‐1375. 10.1101/gr.240663.118 31340985 PMC6673718

[18] Robinson MD , McCarthy DJ , Smyth GK . edgeR: a bioconductor package for differential expression analysis of digital gene expression data. Bioinformatics. 2010;26:139‐140. 10.1093/bioinformatics/btp616 19910308 PMC2796818

[19] Ritchie ME , Phipson B , Wu D , et al. limma powers differential expression analyses for RNA‐sequencing and microarray studies. Nucleic Acids Res. 2015;43:e47. 10.1093/nar/gkv007 25605792 PMC4402510

[20] Chen F , Li Y , Qin N , et al. RNA‐seq analysis identified hormone‐related genes associated with prognosis of triple negative breast cancer. J Biomed Res. 2020;34:129‐138. 10.7555/jbr.34.20190111 32305967 PMC7183303

[21] Vasaikar SV , Straub P , Wang J , Zhang B . LinkedOmics: analyzing multi‐omics data within and across 32 cancer types. Nucleic Acids Res. 2018;46:D956‐d963. 10.1093/nar/gkx1090 29136207 PMC5753188

[22] Scutari M . Bayesian network constraint‐based structure learning algorithms: parallel and optimized implementations in the bnlearn R package. J Stat Softw. 2017;77:1‐20. 10.18637/jss.v077.i02

[23] List M , Amirabad AD , Kostka D , Schulz MH . Large‐scale inference of competing endogenous RNA networks with sparse partial correlation. Bioinformatics. 2019;35:i596‐i604. 10.1093/bioinformatics/btz314 31510670 PMC6612827

[24] Therneau TM , Grambsch PM . Modeling Survival Data: Extending the Cox Model. Springer; 2000.

[25] Yu G , Wang LG , Han Y , He QY . clusterProfiler: an R package for comparing biological themes among gene clusters. OMICS. 2012;16:284‐287. 10.1089/omi.2011.0118 22455463 PMC3339379

[26] Szklarczyk D , Gable AL , Lyon D , et al. STRING v11: protein‐protein association networks with increased coverage, supporting functional discovery in genome‐wide experimental datasets. Nucleic Acids Res. 2019;47:D607‐d613. 10.1093/nar/gky1131 30476243 PMC6323986

[27] Anderson DE , Badzioch MD . Familial breast cancer risks. Effects of prostate and other cancers. Cancer. 1993;72:114‐119. 10.1007/BF00666423 8508396

[28] Overlack K , Primorac I , Vleugel M , et al. A molecular basis for the differential roles of Bub1 and BubR1 in the spindle assembly checkpoint. eLife. 2015;4:e05269. 10.7554/eLife.05269 25611342 PMC4337726

[29] Bolanos‐Garcia VM , Blundell TL . BUB1 and BUBR1: multifaceted kinases of the cell cycle. Trends Biochem Sci. 2011;36:141‐150. 10.1016/j.tibs.2010.08.004 20888775 PMC3061984

[30] Curtis NL , Ruda GF , Brennan P , Bolanos‐Garcia VM . Deregulation of chromosome segregation and cancer. Annu Rev Cancer Biol. 2020;4:257‐278. 10.1146/annurev-cancerbio-030419-033541

[31] Singh AK , Talseth‐Palmer B , McPhillips M , et al. Targeted sequencing of genes associated with the mismatch repair pathway in patients with endometrial cancer. PLoS One. 2020;15:e0235613. 10.1371/journal.pone.0235613 32634176 PMC7340288

[32] Michailidou K , Beesley J , Lindstrom S , et al. Genome‐wide association analysis of more than 120,000 individuals identifies 15 new susceptibility loci for breast cancer. Nat Genet. 2015;47:373‐380. 10.1038/ng.3242 25751625 PMC4549775

[33] Zhang M , Zhao D , Yan C , Zhang L , Liang C . Associations between nine polymorphisms in EXO1 and cancer susceptibility: a systematic review and meta‐analysis of 39 case‐control studies. Sci Rep. 2016;6:29270. 10.1038/srep29270 27387683 PMC4937237

[34] Li Z , Wang Q , Peng S , et al. The metastatic promoter DEPDC1B induces epithelial‐mesenchymal transition and promotes prostate cancer cell proliferation via Rac1‐PAK1 signaling. Clin Transl Med. 2020;10:e191. 10.1002/ctm2.191 33135357 PMC7536616

[35] Chen G , Luo Y , Warncke K , et al. Acetylation regulates ribonucleotide reductase activity and cancer cell growth. Nat Commun. 2019;10:3213. 10.1038/s41467-019-11214-9 31324785 PMC6642173

[36] Wang N , Zhan T , Ke T , et al. Increased expression of RRM2 by human papillomavirus E7 oncoprotein promotes angiogenesis in cervical cancer. Br J Cancer. 2014;110:1034‐1044. 10.1038/bjc.2013.817 24423925 PMC3929894

[37] Wang N , Li Y , Zhou J . Downregulation of ribonucleotide reductase subunits M2 induces apoptosis and G1 arrest of cervical cancer cells. Oncol Lett. 2018;15:3719‐3725. 10.3892/ol.2018.7806 29556274 PMC5844123

[38] Zhong Z , Cao Y , Yang S , Zhang S . Overexpression of RRM2 in gastric cancer cell promotes their invasiveness via AKT/NF‐κB signaling pathway. Pharmazie. 2016;71:280‐284. 10.1691/ph.2016.5843 27348973

[39] Zhang M , Wang J , Yao R , Wang L . Small interfering RNA (siRNA)‐mediated silencing of the M2 subunit of ribonucleotide reductase: a novel therapeutic strategy in ovarian cancer. Int J Gynecol Cancer. 2013;23:659‐666. 10.1097/IGC.0b013e318287e2b3 23466567

[40] Mazzu YZ , Armenia J , Chakraborty G , et al. A novel mechanism driving poor‐prognosis prostate cancer: overexpression of the DNA repair gene, ribonucleotide reductase small subunit M2 (RRM2). Clin Cancer Res. 2019;25:4480‐4492. 10.1158/1078-0432.Ccr-18-4046 30996073 PMC6820162

[41] Liu X , Zhang H , Lai L , et al. Ribonucleotide reductase small subunit M2 serves as a prognostic biomarker and predicts poor survival of colorectal cancers. Clin Sci. 2013;124:567‐578. 10.1042/cs20120240 PMC3562074 23113760

[42] Tate JG , Bamford S , Jubb HC , et al. COSMIC: the catalogue of somatic mutations in cancer. Nucleic Acids Res. 2019;47:D941‐d947. 10.1093/nar/gky1015 30371878 PMC6323903

[43] Demas DM , Demo S , Fallah Y , et al. Glutamine metabolism drives growth in advanced hormone receptor positive breast cancer. Front Oncol. 2019;9:686. 10.3389/fonc.2019.00686 31428575 PMC6688514

[44] Wang W , Cui J , Ma H , Lu W , Huang J . Targeting pyrimidine metabolism in the era of precision cancer medicine. Front Oncol. 2021;11:684961. 10.3389/fonc.2021.684961 34123854 PMC8194085

