# Supplementary material for: A supervised machine learning approach identifies gene‐regulating factor‐mediated competing endogenous RNA networks in hormone‐dependent cancers
Source: J Cell Biochem. 2022 Jun 27;123(8):1394–408. doi: 10.1002/jcb.30300 (PMC9542250; doi:10.1002/jcb.30300)
Supplement: Supplementary file 1 — Supplementary information. [file JCB-123-1394-s001.docx]

**Supporting Information Files**

Supporting Information File 1. List of competing endogenous RNAs found from LASSO-based machine learning models in each hormone-dependent cancer.

| **Cancer** | **List of competing endogenous RNAs** |
| --- | --- |
| PRAD | *ABCC4, ACOT11, ACSF2, ACSL4, ACTC1, ADH1B, AJUBA, AMOT, AMPH, ANKRD33B, ANP32E, ANTXR1, ARHGAP20, ARHGEF26, ARSJ, ASPM, ATAD3C, BDH2, BMP2, BMPER, BNC2, BUB1, BUB1B, C14orf37, C1QTNF1, C8orf88, CAMKK2, CAPG, CAV2, CCDC69, CCDC78, CD200, CDC45, CDCA5, CDCA8, CDH2, CDHR1, CDKN3, CENPF, CENPU, CEP55, CERK, CFH, CHTF18, COCH, COL17A1, COPZ2, CPNE7, CRYAB, CSRNP3, CYSRT1, DBNDD1, DCN, DEPDC1B, DGKG, DIAPH3, DLGAP5, DNAJB4, EFHC2, ELF4, ESPL1, ESRP2, ETNK2, FABP3, FAM107A, FAM169A, FAM83H-AS1, FAT4, FBXO27, FCER1A, FEZ1, FGF10, FHOD3, FILIP1, FILIP1L, FLNA, FLNC, FMO2, FOLH1, FOXA1, FOXM1, FRMD3, FXYD6, GALNT13, GALNT7, GDPD1, GJA1, GMDS, GNAL, GNAZ, GPR124, GPR133, GPR161, GSTM5, HES4, HES6, HJURP, HS3ST3A1, HSF4, HSPA4L, IFI27L2, ISYNA1, ITGB1BP2, ITIH5, JAZF1, KCNB1, KIAA1161, KIAA1244, KIAA1875, KIF18B, KIF20A, KIF4A, LARGE, LMO3, LPCAT2, LRFN1, LRIG1, LURAP1, LYVE1, MACC1, MAP1B, MARCKSL1, MARVELD1, MATK, ME1, MECOM, METTL7A, MKI67, MMRN1, MXD3, MYH11, MYOC, MYOF, MYRIP, NCAM1, NCAPG, NCAPH, NCS1, NEK2, NETO2, NEXN, NHS, NHSL2, NID2, NLRP12, NUF2, OAT, OLFML1, OLFML2A, ONECUT2, OTX1, PAQR8, PARM1, PBK, PCDH9, PDE4A, PDE5A, PDGFC, PDLIM7, PEAK1, PGF, PGM5, PI16, PIK3R1, PKMYT1, PLBD1, PLCL1, PLK1, PLXNA4, PPAPDC3, PPARGC1B, PRIMA1, PRRG4, PTPLAD2, RAB17, RAB38, RAB3B, RAB9B, RALGAPA2, RCAN2, RGS9, RHOB, RND3, RNFT2, ROBO1, RRM2, S1PR5, SCARA3, SCNN1D, SDPR, SEMA3E, SEMA5A, SH3RF1, SHCBP1, SIX2, SKA3, SLC16A7, SLC25A45, SLC35F1, SLC38A5, SLC40A1, SLC7A4, SLFN12, SLIT3, SNX7, SORCS1, SOX15, SPAG5, SPTBN2, SPTLC3, ST5, STIL, SV2A, SYNC, TIMP4, TINAGL1, TK1, TLCD1, TLR2, TMEM246, TMEM35, TMOD1, TMTC4, TOP2A, TPRG1, TRIM22, TRIP13, TROAP, TRPC6, TTK, UBE2C, UNC5B, WNT2B, ZGLP1, ZMYND10,* lncRNA-ADAMTS9-AS1*,* lncRNA-MAGI2-AS3*, PDLIM5, ARHGEF40, PINLYP,* lncRNA-LINC00900*, ITGB3, SMIM10, KANK2, SCNN1B,* lncRNA-ADAMTS9-AS2*,* lncRNA-LINC01503*, IQGAP3, SLC12A8,* lncRNA-BOLA3-AS1*, FABP5,* lncRNA-LINC00665*,* lncRNA-ACTA2-AS1*, KIFC1, AURKB, C4orf48, SLC25A22,* lncRNA-BCRP3*, FAM46A,* lncRNA-SRRM2-AS1*, PTTG1, CTHRC1, PCDHGB1,* lncRNA-LINC01018*, GSTM4, SBK1, MYBPC1, EPHA5,* lncRNA-CRNDE*, POU5F1B, KCNK3, SHISA9, TMEM52, SAPCD2, CDK5R1, SLIT1, TDRD6, C1orf64, NAGS, ERBB4* |
| BRCA | *ABCA1, ABCG2, ABRACL, ACACB, ACER2, ACKR4, ACOT7, ACSL4, ACSM3, ACTA2, ACVR1C, ADAM12, ADAM19, ADAM33, ADAMDEC1, ADAMTS14, ADAMTS3, ADAMTS4, ADAMTS6, ADAMTS9, ADAP1, ADTRP, AHNAK, AIF1L, AKAP3, AKR1C2, AKT3, ALDH1A1, ALDH1A2, ALDOA, ALG3, ALS2CL, AMOTL1, ANGPT1, ANGPTL2, ANKRD30A, ANLN, ANO6, ANTXR2, ANXA1, ANXA3, ANXA9, AP1S3, APBA1, APBB1IP, APCDD1L, ARF1, ARHGAP10, ARHGAP26, ARHGAP28, ARHGEF39, ARHGEF4, ARHGEF6, ARID5B, ARPC1B, ARRDC3, ASPN, ATOH8, ATP2B4, ATP6AP1, ATP6V0B, AUNIP, AURKA, B4GALT6, BACH2, BAX, BBOX1, BCAS1, BCL11A, BCL2L12, BGN, BICC1, BMPR1B, BTNL9, BUB1, BUB1B, C10orf128, C17orf51, C1QTNF6, C1orf112, C20orf194, C22orf23, C2orf88, C3, C3orf58, C3orf80, C9orf116, CA12, CAB39L, CABLES2, CACFD1, CACNA1D, CALCOCO1, CAPN13, CAPN9, CARD6, CASKIN1, CASQ2, CBX2, CBX7, CCDC137, CCDC150, CCDC158, CCDC24, CCDC69, CCDC82, CCNA2, CCNB1, CCNB2, CCNE1, CCNE2, CCNF, CCT3, CD209, CD248, CD3EAP, CD93, CDC14A, CDC20, CDC25A, CDC7, CDCA2, CDCA3, CDCA4, CDCA5, CDCA8, CDH11, CDH13, CDH2, CDK1, CDKN2A, CDS1, CEACAM5, CEMIP, CENPE, CENPF, CENPI, CENPL, CENPM, CENPN, CENPO, CENPU, CEP55, CEP68, CEP85L, CERS2, CFL1, CFP, CH25H, CHAF1A, CHEK1, CHL1, CHRM3, CHST11, CHST3, CHST6, CHST9, CHTF18, CIT, CKAP2L, CLCN4, CLEC10A, CLIC2, CLIP4, CLTCL1, CMTM7, CMYA5, CNN1, CNRIP1, CNTN1, CNTN3, COBLL1, COL10A1, COL11A1, COL12A1, COL1A2, COL22A1, COL3A1, COL4A4, COL5A1, COL5A2, COL8A1, COMP, CORIN, CPE, CREB5, CREBRF, CRIM1, CRY2, CRYAB, CRYBG3, CSMD2, CSRNP1, CSRNP3, CTPS1, CUBN, CXCL9, CYB561, CYBRD1, CYP26B1, CYP4B1, CYP7B1, CYR61, DAB2, DBF4, DCAF13, DCLK1, DCN, DDIAS, DDX39A, DEGS2, DEPDC1, DEPDC1B, DIAPH2, DIAPH3, DIXDC1, DLG3, DLGAP5, DNA2, DNAJB4, DNMT3B, DOCK11, DOPEY2, DPYD, DSC3, DSCC1, DSEL, DSG3, DTYMK, DUSP6, DYNC2H1, E2F2, E2F5, E2F8, EBF1, ECE2, ECM2, ECT2, EDA2R, EDNRB, EFNA3, EHBP1, EHD2, EIF4E3, EME1, EME2, EMILIN2, ENPP2, ENTPD7, EOGT, EPAS1, EPB41L2, EPDR1, EPHB2, ERBB3, ERCC6L, ESCO2, ESRP1, ESYT3, ETV1, EXO1, EZH2, F10, F13A1, F7, F8, FAM102A, FAM124A, FAM129A, FAM135A, FAM171A1, FAM47E, FAM49A, FAM83D, FANCA, FANCD2, FAT1, FAT2, FAT3, FBN2, FBXL19, FCN1, FERMT1, FERMT2, FGD3, FGF7, FGL2, FKBP4, FLAD1, FLI1, FMO1, FN1, FNDC1, FNDC4, FOLH1, FOXA1, FOXM1, FOXN3, FOXP3, FRMD3, FRY, FTO, FXYD6, GABRE, GALE, GALNT15, GALNT7, GAS1, GAS2L3, GATA3, GBP5, GDF15, GDPD5, GINS1, GINS2, GINS4, GJC2, GLIPR1L2, GNAI1, GNG2, GPAM, GPM6B, GPR34, GPR68, GPRIN1, GPSM2, GRHL2, H2AFZ, HABP4, HBEGF, HCAR1, HDGF, HELLS, HES6, HGF, HIST1H2BJ, HJURP, HMGA1, HMGB3, HMMR, HNRNPAB, HOMER3, HOOK1, HOXD9, HPRT1, HSD17B6, HSPE1, IFFO2, IGF2BP2, IGFALS, IL11RA, IL1R1, IL1RL2, IL34, IL4I1, INHBA, INTS7, IQGAP3, IRF4, ISM1, ITGA7, ITGA9, ITGB8, ITPR1, KALRN, KANK4, KAT2B, KCNC4, KCND3, KCNE4, KCNIP2, KCNK6, KCNMA1, KCNQ4, KCTD12, KIF11, KIF12, KIF14, KIF15, KIF18A, KIF18B, KIF20A, KIF23, KIF26A, KIF26B, KIF2C, KIF4A, KIT, KLF10, KLF5, KLHDC1, KLHDC9, KLHL21, KLHL29, KLK10, KLK7, KLRB1, KNOP1, KNSTRN, KPNA2, KRT16, KRT18, KRT5, KRT6B, L3MBTL4, LAD1, LAMB3, LAMP3, LAMP5, LEPR, LGALS3, LIF, LIG1, LIMK1, LIPE, LLGL2, LMNB2, LMO2, LONRF3, LPAR6, LPIN1, LRFN1, LRFN4, LRIG3, LRP1, LRP8, LRRC15, LRRC26, LRRC59, LRRK2, LRWD1, LTBP4, LYVE1, MAD2L1, MAGED2, MAGI1, MAL2, MAML2, MAMLD1, MANF, MAP2, MAP7D3, MARCO, MARVELD2, MATN3, MAZ, MBNL2, MBNL3, MCAM, MCC, MCM10, MCM2, MCM4, MCTP1, MCTP2, MDFIC, MELK, MEOX1, MFGE8, MGAT3, MICAL2, MICALL2, MID1, MITF, MKI67, MLPH, MME, MMP11, MMP13, MMP14, MMP7, MMP9, MND1, MOB3B, MPP6, MRAS, MRPL14, MRVI1, MSR1, MSRB3, MTFR2, MTHFD2, MTMR10, MUC15, MUC16, MYB, MYBL1, MYBL2, MYCBP2, MYCT1, MYH3, MYLK4, N4BP3, NALCN, NANS, NAT1, NCAPG, NCAPH, NCMAP, NCOA7, NDC80, NECAB3, NEDD9, NEIL3, NEK10, NFAT5, NFIB, NFIL3, NGFR, NIPAL1, NMT2, NOV, NOVA1, NREP, NT5E, NTN4, NUDT11, NUDT16L1, NUSAP1, OAS2, OLFML2B, OMD, ORC1, ORC6, OSR1, OTX1, P2RY13, P2RY14, PAFAH1B3, PARD3B, PARD6B, PARP1, PBK, PCBP3, PCDH17, PCDH19, PCYOX1, PDE1A, PDE1C, PDGFD, PDIA4, PDK4, PDLIM4, PELI1, PFDN6, PGR, PHLDB1, PIF1, PIK3R1, PIWIL4, PKMYT1, PKP1, PLA2R1, PLAGL1, PLAUR, PLD1, PLEKHD1, PLEKHG1, PLIN1, PLIN2, PLIN4, PLK1, PLK4, PLSCR4, PLTP, PM20D2, PNPLA2, POLQ, POSTN, PPARA, PPARGC1B, PPEF1, PPM1F, PPM1L, PPP1R37, PPP4C, PRDM5, PRDM8, PREX2, PRKCA, PRKG2, PRLR, PROX1, PRR11, PRR15, PRRG1, PRRT3, PRSS12, PRX, PSAT1, PSRC1, PTCH1, PTGER3, PTK6, PTK7, PTPN14, PTPN21, PTPRG, PTTG1, PTX3, PUSL1, QKI, RAB17, RAB19, RAB31, RAB6B, RABEP2, RACGAP1, RAD51, RAD51AP1, RAD54B, RAD54L, RAI2, RAPGEF3, RARRES1, RARRES2, RASEF, RASGRF2, RASGRP2, RBMS2, RCAN1, RCC1, RCC2, RECQL4, REEP4, RELL2, RELT, RET, RFC4, RGS5, RHOF, RHOQ, RIMS3, RIMS4, RMI1, RMI2, RNASEH2A, RNF125, RNF217, RNFT2, ROCK2, ROGDI, ROR1, RPS6KA2, RPS6KA3, RPUSD1, RRAGD, RRM2, RSAD2, RSPH1, RTKN, RTN1, RTN4R, RUNX2, RYR3, SACS, SALL4, SAMD14, SAMD4A, SAPCD2, SCN7A, SDC1, SDSL, SEC31B, SEMA3C, SEMA3D, SEMA3G, SEMA5A, SEMA7A, SH2D2A, SH3D19, SHC4, SHCBP1, SIGIRR, SKA1, SLAIN1, SLC16A3, SLC16A6, SLC16A7, SLC19A3, SLC1A3, SLC22A18, SLC22A3, SLC24A2, SLC25A27, SLC25A37, SLC2A1, SLC2A12, SLC2A6, SLC35A2, SLC35G1, SLC38A5, SLC52A2, SLC6A16, SLC7A5, SLC9A9, SLIT2, SLIT3, SMS, SNAI2, SNRNP25, SNRPB, SOCS2, SORBS1, SORD, SOX11, SOX6, SPAG1, SPAG5, SPARCL1, SPC24, SPC25, SPOCD1, SPTBN1, SSPN, SSX2IP, ST14, ST3GAL6, ST6GAL2, ST8SIA1, STAC, STAC2, STARD8, STEAP2, STEAP4, STIL, STMN1, STOX2, STRBP, STS, STX11, SULF1, SV2B, SYNDIG1, SYNE1, SYNM, SYT8, TACC3, TAP1, TBC1D4, TCF7, TCF7L1, TDO2, TDRD6, TEAD1, TFCP2L1, TGFBR2, THRB, THSD1, TICRR, TIMELESS, TIMM17A, TIMM17B, TIPARP, TLCD1, TLL1, TLR3, TLR4, TMCC3, TMEM132A, TMEM145, TMEM184A, TMEM206, TMEM246, TMEM63B, TMEM8A, TMEM97, TMOD1, TMOD2, TNFRSF21, TNFRSF4, TNFRSF9, TNS1, TNS2, TOMM40, TONSL, TP63, TPD52, TPM2, TPM3, TPX2, TRABD2A, TRAIP, TRIM2, TRIM29, TRIP13, TRPS1, TSPAN1, TSPAN11, TSPAN13, TTC28, TTC39A, TTK, TTYH1, TTYH3, TUBA1C, TUBB6, TUBG1, TUSC5, TYMP, TYMS, TYRO3, UBE2C, UBE2T, UGP2, UGT8, UNC5B, USP53, UTRN, VCAN, VIM, VSNL1, VWDE, WDR86, WISP1, WLS, WNT11, YBX3, YDJC, ZBTB16, ZBTB20, ZC3H12C, ZC4H2, ZCCHC24, ZEB2, ZFP36, ZNF367, ZNF467, ZNF540, ZNF552, ZNF711, ZWILCH, ZWINT, MAP3K5, CD302, GCNT4, IGIP, AKAP5, PRC1, RCOR2, MICAL3, RFTN2, ITGA1, ZNF469, PEAR1, TWIST2, SERPINB5, PHLDB3, PPP1R14C, ABLIM3, DCUN1D3, AKR1C1, COL6A6, TIGIT, MB, KIFC1, FADS3, RELN, B3GNT5, TRIM59, ZNF695, ASPH, DPP4, CANT1, APOBEC3C, RABIF, SH3RF2, TLN2, PERM1, GIMAP1, GNG7, GABRD, TSEN54, MEX3A, PLEKHM3, ADAT3, CHRM1, RGS19, GPX3, LRRC73, HID1, ARHGAP11A, CX3CR1, PAX9, LAMA2, LDB3, GSTM2, LIME1, NME1, CCDC167, GPC2, BRI3BP, SCG2, HGH1, EBF2, MMP1, KLF11, PKDCC, NAP1L2, ETV5, FAM89A, RYR1, PPP1R3G, CPNE7, PTPRM, CLEC5A, C5orf46, JAM3, TMEM170B, GIMAP5, ABHD12, F3, TCP11L2, EVL, FAM222A, C4orf48, FZD4, HYAL3, ACSM5, NR3C2, PDE7B, TNFSF4, ADRA2A, KPNA5, CALB2, NIPSNAP3B, GJB2, SLC29A4, TUBB2B, PLIN5, H2AFX, SHANK3, GLDN, FAM43A, MRGPRF, HCAR2, ATP8A1, ANKDD1A, TMPRSS6, GRAMD1A, GFOD1, PLCG2, TMEM178B, ZNF703, OSCAR, CEP112, GOLM1, FAM196A, ADM, SOCS3, CTIF, F2RL2, ILDR2, MXD3, APOO, IRS1, SPINT1, KIF24, DLK2, MYC, AMOTL2, KCNB1, NXPH4* |
| COLCA | *A2M, ABCC9, ABHD5, ACAN, ACTR3B, ADAM12, ADAM33, ADAMDEC1, ADAMTS12, ADAMTS2, ADAMTSL1, ADAMTSL3, ADAT2, ADH6, ADTRP, AGR3, AHNAK, AKAP5, ALDH1L1, ALDH6A1, ANGPT2, ANKRD12, ANKRD44, ANLN, AOX1, APOBR, AQP9, ARHGAP10, ARHGAP28, ARHGAP31, ARHGEF19, ARHGEF39, ARHGEF6, ARID3A, ARMC10, ASAP3, ASCL2, ATAD2, ATIC, ATOH1, ATP11A, ATP8B4, AUNIP, AURKA, AURKB, AXL, BCAS1, BCL2, BHLHE41, BIRC5, BRSK2, BUB1, BVES, C17orf53, C2orf88, C4BPB, C7, CA1, CACNA1D, CACNA2D2, CAD, CADM1, CALB2, CAMTA2, CASP7, CAV2, CBX7, CCDC85B, CCL13, CCL28, CCM2L, CCNA2, CCNB1, CCNB2, CCNE1, CCNF, CCR2, CCT2, CD109, CD14, CD163, CD163L1, CD209, CD300LF, CD36, CD3D, CD3EAP, CD48, CD53, CD86, CDC45, CDCA2, CDCA5, CDCA8, CDHR5, CDK2, CDKN2B, CDKN3, CEMIP, CENPE, CENPH, CENPK, CEP55, CEP72, CERS4, CHEK1, CHGA, CHP1, CHRNA3, CHTF18, CITED2, CKAP2L, CKS2, CLEC10A, CLMN, CLSPN, CMKLR1, CMSS1, CNIH3, CNN1, COL10A1, COL11A1, COL12A1, COL5A1, COL5A2, COL7A1, COPZ2, CPA3, CPEB2, CPLX2, CPT2, CPXM1, CSE1L, CTSE, CYP1B1, CYSLTR1, DAAM1, DBF4, DBF4B, DCLK2, DCUN1D5, DDIAS, DDX11, DDX21, DDX56, DEPDC1B, DGKA, DHDDS, DHRS9, DIAPH3, DIRC2, DKC1, DLGAP5, DNAJC2, DOCK2, DPEP1, DPH2, DSC2, DSCC1, DTNA, DTX1, DUS1L, DUS4L, DUSP1, DUSP4, E2F1, E2F7, ECE2, EDNRB, EIF2S2, ELOVL6, EME1, EMP1, ENTPD5, ENTPD8, EPB41L4B, EPHA3, EPHA4, ESPL1, EVC, EXOSC2, EZH2, F12, F13A1, FAM126A, FAM126B, FAM13C, FAM214A, FAM46A, FAM72A, FANCB, FANCI, FAS, FBLN1, FBXL5, FBXO32, FCGR2B, FCGRT, FECH, FEN1, FGFR2, FHL1, FKBP5, FLNA, FLNC, FMO4, FNIP2, FOLR2, FOXN3, FRMD4B, FRYL, FSTL3, FZD4, GAB1, GABARAPL1, GALNT15, GALNT8, GARS, GIMAP6, GINS1, GINS2, GLIPR2, GNAQ, GNG7, GPR65, GPSM2, GPT, GRPEL2, GTF2IRD1, GTSE1, HAUS6, HECW2, HELLS, HES6, HHIP, HILPDA, HLA-DMB, HM13, HMGB3, HSD17B6, HSPB7, HSPB8, HSPH1, IARS, IFIT2, IL16, ISG20, ITGA4, ITGA7, ITGA8, ITGB7, ITPR1, KANK2, KCNAB1, KCTD12, KIAA0513, KIF14, KIF18B, KIF2C, KIF4A, KIF5C, KIF9, KIT, KITLG, KLF2, KLF4, KLHL31, KLHL5, KLK10, KNSTRN, KPNA2, KRT23, LAS1L, LAX1, LCN12, LHFPL2, LIMA1, LIMCH1, LMNB2, LOXL2, LRRC15, LRRC25, LXN, LY9, LYAR, LYVE1, LZTS1, MAB21L2, MAGI3, MAP1B, MARCO, MATN3, MCM10, MCM2, MCM3, MCM4, MCM6, MEIS2, MELK, MEOX1, MEP1A, MFAP2, MGAT4A, MGLL, MKI67, MMP11, MPP7, MS4A12, MS4A4A, MS4A6A, MSI2, MTERF3, MTFR2, MTHFD1L, MTHFD2, MTMR9, MUC4, MUC5AC, MYCT1, NAAA, NCAPD3, NCAPG, NCAPH, NCKAP1L, NDUFA5, NEDD9, NEIL3, NEK1, NELFCD, NEURL1, NFATC1, NOP2, NOP58, NOX4, NPEPL1, NPR3, NPTN, NR3C2, NR6A1, NRP2, NRXN3, NUCB2, NUDCD1, NUSAP1, ODC1, OLFML1, OLR1, ORC6, P2RX1, P2RX4, P3H1, PABPC1, PAFAH2, PALD1, PALLD, PALMD, PAMR1, PANK3, PARPBP, PCDH19, PCNA, PCSK7, PDCD11, PDCD4, PDE1A, PDE3B, PDE5A, PDLIM2, PDLIM7, PFDN4, PGF, PHLPP2, PIK3CG, PLA2G4A, PLA2G4F, PLCE1, PLCL1, PLCL2, PLD4, PLK1, PLK4, PLOD2, PLSCR4, PLXNC1, PMFBP1, PNO1, PNPT1, POLE2, POLR1A, POLR3G, POP1, POU2AF1, POU5F1B, PPAT, PPP1R3C, PRDM8, PRMT1, PRR11, PRRX1, PRSS12, PSRC1, PTBP2, PTGDS, PTGER2, PTGER3, PTGS1, PTP4A3, PUS7, PVR, R3HDML, RAB27A, RAB30, RAB34, RAD51AP1, RAD54L, RAE1, RANBP1, RAP1A, RASD2, RASGRP2, RASL12, RASSF6, RASSF8, RBM28, RBM47, RBMS3, RCC1, RECQL4, REEP2, RFC3, RIMS3, RNASEL, RNF11, RNF125, RNF152, RNF43, ROR1, RPN2, RPS6KA5, RRM2, RRP12, RRP9, RRS1, RTKN, RUVBL2, SALL4, SAPCD2, SCARB1, SCML1, SCN4B, SCUBE2, SDC2, SEMA6A, SERPINE1, SFRP1, SFRP4, SGCB, SGK2, SGMS2, SHROOM3, SHROOM4, SIAE, SIDT2, SIPA1L2, SKA1, SKA3, SKP2, SLAMF1, SLC11A1, SLC12A9, SLC13A3, SLC16A7, SLC24A3, SLC25A24, SLC29A1, SLC35G1, SLC36A1, SLC5A6, SLC7A2, SLC9A9, SLIT2, SLITRK6, SMCHD1, SMIM14, SMPD3, SORD, SPAG5, SPARC, SPC25, SPDEF, SPDL1, SPECC1, SPOCD1, SPP1, SRM, ST3GAL3, ST3GAL4, ST8SIA4, STAB1, STARD8, STARD9, STPG1, STRIP2, STX11, SULF1, SUSD6, SUV39H1, SUV39H2, SYNM, SYNPO2, TAGLN, TBC1D30, TCF21, TCF7, TCN1, TCOF1, TELO2, TFCP2L1, THOP1, THSD4, TICRR, TIMELESS, TLR1, TMEM131, TMEM140, TMEM161A, TMEM236, TMEM97, TNFSF9, TNS1, TONSL, TOP2A, TPX2, TRABD, TRIB3, TRIM28, TRIP13, TROAP, TRPC1, TSPYL5, TTC26, TTC28, TTI1, TTK, TTLL4, TTYH2, TUBA1A, UBE2T, UNC5B, URB1, URB2, UST, UTP14A, VAV3, VCL, VLDLR, VNN2, VPS4B, VSIG2, WASL, WDFY4, WDR12, WDR18, WDR43, WDR62, WDR7, WDR74, WDR78, WFDC1, WISP1, WNT5A, WNT9A, XPO5, YDJC, YPEL5, YTHDF1, ZBTB4, ZDHHC9, ZEB1, ZEB2, ZNF74, ZZEF1,* lncRNA-SATB2-AS1*,* lncRNA-SNHG12*, FAT4, ST6GALNAC3, ZNF788, GJC1, ADAMTS4, ZNF469,* lncRNA-SNHG11*, PBX3, SERPINH1, GPC6, FJX1, LAYN, CYS1, MSH5, C11orf86, FAM114A1, CHST6, MTMR10, DPF3*, lncRNA-MUC2*, TMEM30B, CLEC5A, GCSAM, SYNJ2BP, CSF1, BLM, AQP1, RCN3, C10orf128, PRKACB, GPR34, TLR7, TOP1MT, PGAM5, WDHD1, NKRF, NOTUM, CHD7, TAMM41, RCBTB1, SLC7A1, RUVBL1,* lncRNA-PCED1B-AS1*, PPIL1, COL4A1, FER1L6, F8, CD177, SPTBN2, GPX3, KIF13B,* lncRNA-DNM3OS*, MYOM1, C2CD4D, DNASE1L3, CENPW, MAD2L1, PRC1, FANCA, FAM46C, CDCA4, C1QC, ZNF695, DTYMK, EXO1, MAB21L3, PSAT1, XRCC2, BUB1B, STRA6, DGKZ, VARS, XPOT, SSC5D, FAM229B, SUCLG2, NCKAP5, SERPINB5, RNASE4, FFAR4, LDHD, C4orf19,* lncRNA-DLEU2*, PDF, RNF138, DMD, TFAP2A, PTTG1, CHCHD6, MCM7, LDLRAD3, BDKRB2, CKB, ZFP28, FASN, WNT2, CSF1R, ZNF25, GALNT1, SYNE3, REEP3, XPNPEP2,* lncRNA-LINC00654*, DPYD, TRAIP, CPNE1, ERGIC3, PLOD3, PSPH, TINAG, DTL, PCID2, CIDEC, SLC26A2, CAPN2, TIGD1, DSG3, ARSJ, LITAF, ECT2, PPARGC1B, ARHGEF38,* lncRNA-PVT1*, IQGAP3, HK2, UNC5C, ANKRD13B, RAD54B, VAPA, EGFL6, AMN, FAM49A,* lncRNA-SNHG6*, KIFC3, TRANK1, COX7A1, CEP250,* lncRNA-LINC01315*, THBS2, EFNA5, JADE3, H2AFX, CCNG2, CTSS, DNAAF5, GCNT3, GDPD5, TLN2,* lncRNA-LINC00174*,* lncRNA-VIPR1-AS1*, QSOX2, L1CAM, SOWAHA, TMEM56, PUS1, DENND1C, GALK1, TRMT1, DDIT4, MFSD12, PPRC1, TWIST2, TRPC6, GFRA1, SLC22A23,* lncRNA-SNHG3 |
| UCEC | *ABCA7, ABL1, ADAMDEC1, ADCY3, ADCY9, AEBP1, ALDOA, AMOTL2, ANKLE1, ANKRD33B, ANKRD44, ANLN, APBB1, APOD, APOL3, ARHGEF16, ARHGEF39, ARHGEF6, ARL10, ARSI, ASPN, ATAD3A, ATAD5, ATP11B, ATP2C2, ATP8B4, AUNIP, AURKA, AURKB, AXL, B4GALNT3, BCAP29, BEND3, BIK, BLM, BOLA3, BRCA1, BRI3BP, BRIP1, BUB1, BUB1B, C17orf53, CADPS2, CCDC134, CCDC150, CCDC18, CCNB1, CCNE1, CCNF, CD22, CD300LF, CD40, CD44, CD99L2, CDC6, CDC7, CDCA2, CDCA3, CDCA5, CDCA8, CDCP1, CDHR3, CDK1, CDKL5, CDKN1B, CDKN2A, CDON, CELSR3, CENPA, CENPE, CENPK, CENPL, CENPM, CENPN, CEP55, CFL2, CHAF1B, CHEK1, CKAP2, CKAP2L, CKS1B, CLCN2, CLDN1, CLIC4, CMKLR1, CNRIP1, COL21A1, COL3A1, COL4A4, COL5A1, COMP, CP, CPVL, CREBL2, CSGALNACT1, CSPG5, CTSV, CXCL11, CXorf36, CYB561, CYBA, DAB2, DARS2, DCDC2, DDX11, DEPDC1, DEPDC1B, DHCR24, DHCR7, DLGAP5, DNA2, DNAJB11, DNAJC18, DNMT1, DNMT3B, DPP4, DSCC1, DSN1, DTWD1, DTYMK, E2F2, E2F8, ECE2, ECT2, EMCN, EME1, ENO1, ENPP5, ENTPD1, EPAS1, EPB41L3, EPHA4, EPS8, ESCO2, ESPL1, ESRP1, EVC, EXO1, EZR, FAM126A, FAM136A, FAM83D, FAM83E, FANCB, FANCI, FAS, FASN, FBN1, FBN2, FCHO1, FEN1, FGFBP1, FGFR4, FLNA, FMOD, FOXK1, FOXM1, FOXN3, FRAS1, FRMD6, FSTL3, FUT2, FZD7, GAB2, GABPA, GAD1, GALE, GALNT3, GAPDH, GAS2L3, GCNT2, GDA, GDF7, GGCT, GGH, GIMAP2, GIMAP6, GINS1, GINS2, GLI1, GLIPR1, GLIS2, GNA14, GNG4, GPI, GPR84, GPRC5A, GTSE1, GUCY1A2, GZMK, H2AFY, H2AFZ, HAUS8, HDGF, HEBP1, HERC5, HES2, HHEX, HIF3A, HJURP, HK3, HKDC1, HMGA2, HMGB3, HNRNPAB, HSPE1, ICAM2, IL15RA, IL16, IL17RD, ILF2, INPP5A, INSR, IQGAP3, IRS2, ITGA11, ITGAV, KANK1, KCNK6, KHK, KIF15, KIF18A, KIF18B, KIF20B, KIF22, KIF4A, KLF11, KLF6, KNSTRN, KNTC1, LAMC2, LDHA, LDLRAD4, LIG1, LLGL2, LMNB2, LPCAT2, LRCH1, LRP1, LRP12, LRP8, LRRC3, LSM4, LTBP4, MAD2L2, MAP3K9, MARVELD1, MAST1, MASTL, MB21D2, MCAM, MCM10, MCM2, MCM4, MCM8, MELK, MFAP2, MGME1, MISP, MKI67, MMP11, MMP15, MND1, MPRIP, MRPL15, MRPS34, MSRB3, MTFR2, MTHFD1L, MTHFD2, MUC20, NCAPD2, NCAPD3, NCAPG, NCAPG2, NCMAP, NDC80, NEK1, NEK2, NFE2L3, NLGN2, NME1, NOP16, NOP2, NPR1, NRIP2, NT5DC2, NT5E, NUAK1, NUDT5, NUF2, NUP210, NUSAP1, OAZ3, ORC1, ORC6, OVGP1, P2RY6, P3H2, P3H3, PACRG, PAMR1, PARPBP, PCK2, PCNA, PDLIM5, PDSS1, PGP, PHLPP2, PIF1, PLCE1, PLEKHH1, PLEKHS1, PLXNC1, PNKD, POC1A, POLE, POLE2, POLQ, POSTN, PPM1F, PPM1G, PPP1R12A, PPP1R13L, PRC1, PRR11, PRRX1, PRSS22, PRX, PSAT1, PSMD14, PSRC1, PTBP2, PTK6, PTPN6, PTPRB, QKI, RAB11FIP2, RAB31, RACGAP1, RAD51AP1, RAD54L, RAP1A, RASAL1, RASD2, RBFOX2, RBPMS, RECQL4, RELT, RFC4, RFTN1, RHBDF2, RIMS3, RNF144A, RNF43, ROBO4, RPS6KA1, RPS6KA5, RRAD, RRM2, S1PR1, SACS, SALL2, SAPCD2, SCD, SCML2, SEC23A, SEL1L3, SETD7, SFRP4, SGTB, SH2D3A, SHE, SHMT2, SIGLEC1, SKA1, SKA3, SKP2, SLC12A4, SLC22A18, SLC23A2, SLC25A13, SLC27A4, SLC2A3, SLC35G2, SLC4A11, SLC52A3, SLC5A1, SLC5A6, SLC8A1, SLCO2B1, SMAD3, SMG6, SMPDL3B, SNED1, SNRNP25, SOCS5, SPAG5, SPC25, SPEG, SPON2, ST13, STMN1, STXBP1, SYNGR3, SYT13, SYTL1, SYTL4, TACC3, TAGAP, TBC1D2B, TFRC, TGFB1I1, THOC3, TICRR, TIMELESS, TIMM10, TKT, TLR4, TMC5, TMEM136, TMEM184A, TMEM206, TMEM245, TMEM63B, TMEM97, TMOD2, TNFRSF9, TNNT1, TNS2, TNXB, TOMM40, TONSL, TOP2A, TPD52, TPI1, TPPP3, TPX2, TRABD, TRAIP, TRIB3, TROAP, TSPAN1, TSPAN5, TTC9, TTK, TTYH3, TUBA1C, TUBG1, TUFT1, TWSG1, UBE2S, UST, VCL, WDHD1, WDR62, WFS1, WNT10A, WNT7A, XDH, XRCC2, ZBTB4, ZCCHC24, ZNF519, ZNF695, ZNF704, ZWILCH, ZWINT, ZYG11B, TNFSF12, APOBEC3G, NPTXR, SMTN, MAP3K3, VDAC1, TSPAN4, ACKR1, AGPAT2, NOTCH4, BBS10, STMN3, HMBS, DHFR, RALGPS2, RPS6KA3, HES6, TRANK1, ARHGAP11A, RTKN2, KPNA2, VARS, ERICH3, KDELC2, PGAM5, UQCRQ, SOWAHC,* lncRNA-TMPO-AS1*, CDC25A, GPR137C, NRIP3, IGF2, C1R, ST3GAL3, TPPP, BOC, RRAGB, FAM222A, PBK, MTFP1, DENND5B, ARL4C, MAF, GIMAP1, GREM2, GNG2, CYS1, HPSE, PPP1R3B, PAQR4, GRIN1, SPATA17, TSPAN13, TAPBP, C12orf75, RAC3,* lncRNA-HAND2-AS1*, ENTPD7, CLDN7, B3GNT3, GULP1, TBC1D7, UCHL3, CAPN8, KLHDC7A, CDH2, SBK1, PGBD5, TUBB3, ROR2, AHNAK2, FAT4, FRMD5, STON1, ZHX1, GCNT1, SLCO2A1, TMEM150C, EPCAM, PIK3R3, NDNF, SLC9A9, STARD5, RCC2, HOPX, SERPING1, MS4A8, PLAG1, SLC25A33, DRAXIN, TACC1, TLN1, BCL2L15, SGK1, SLC37A1, GPR35,* lncRNA-COLCA1*, ATP8B2, PRKD1, NMT2,* lncRNA-BOLA3-AS1*, EZH2, CBX2, FSTL1, NIPSNAP1, SERPINA1, MRAS, TMEM43, CEP85, ARMC4, FAM189A2, PELI3, FYCO1, FANCA, MCM7, ARRDC1, ADM2, DUS1L, CD93, BMP2, RMI2, PBX3, SEC23B, SPINT1, CA8, CYP4X1, TGM2, CTNNAL1, ABCA3, KCTD7, TMEM63A, HECW2, NEU1, PNRC1, PTCH1* |
| PRAD∩BRCA | *BUB1B, CDCA5, CDCA8, CENPF, CENPU, CEP55, CHTF18, CRYAB, CSRNP3, DCN, DEPDC1B, DIAPH3, DLGAP5, FOXM1, FXYD6, HJURP, IQGAP3, KIF18B, KIF20A, KIF4A, LRFN1, MKI67, NCAPG, NCAPH, PBK, PKMYT1, PLK1, PTTG1, SEMA5A, SHCBP1, SLIT3, SPAG5, TRIP13, TTK, UBE2C* |
| PRAD∩COLCA | *CDKN3, DEPDC1B, NCAPH, OLFML1, NCAPG, BUB1, RRM2, DIAPH3, FAT4* |
| PRAD∩UCEC | *BUB1B, CDCA5, CDCA8, DEPDC1B, HJURP, MKI67, NEK2, SPAG5, TOP2A, BUB1, AURKB, RRM2, DLGAP5, NUF2, IQGAP3, KIF18B* |
| BRCA∩COLCA | *AHNAK, ANLN, AURKA, BUB1, CCNB1, CCNB2, CCNE1, CD209, CDCA2, CDCA5, CENPE, CHEK1, CHTF18, COL10A1, COL12A1, DBF4, DEPDC1B, DIAPH3, EME1, EZH2, FOXN3, GINS1, KCTD12, KIF14, KIF2C, KIF4A, KNSTRN, MATN3, MCM2, MELK, NCAPG, NCAPH, NEIL3, NUSAP1, ORC6, PLK4, PLSCR4, PRR11, PSRC1, RECQL4, SKA1, TICRR, TTK, UNC5B, MTMR10, CCNF, AUNIP, SAPCD2, MAD2L1, RCC1, C10orf128, ZNF695, DTYMK, FANCA, LMNB2, SULF1, ZNF469, TONSL, ARHGEF6, SLC9A9, BUB1B, RAD51AP1, CCNA2, DDIAS, LRRC15, RRM2, SPC25, DLGAP5, CKAP2L, ITPR1, EXO1, RAD54B* |
| BRCA∩UCEC | *ANLN, AUNIP, AURKA, BUB1, BUB1B, CCNB1, CCNE1, CCNF, CDC7, CDCA3, CDCA5, CDCA8, CENPE, CENPL, CENPN, CEP55, CHEK1, CKAP2L, DEPDC1, DEPDC1B, DLGAP5, DNA2, DSCC1, DTYMK, E2F2, ECE2, EME1, ESCO2, FAM83D, GAS2L3, GINS1, GINS2, HJURP, HMGB3, KIF15, KIF18A, KIF4A, KNSTRN, LIG1, LMNB2, MCM10, MCM2, MCM4, MELK, MKI67, MND1, MTFR2, NCAPG, ORC1, ORC6, PIF1, POLQ, PRR11, PSRC1, RACGAP1, RAD54L, RECQL4, RELT, SKA1, SPAG5, STMN1, TACC3, TICRR, TIMELESS, TOMM40, TONSL, TPX2, TTK, ZWINT, EXO1, ZNF695, ZWILCH, KPNA2, SPC25, HNRNPAB, CDCA2, PRC1, SAPCD2, TUBA1C, TRAIP, NME1, BRI3BP, RRM2, IQGAP3, RCC2, E2F8, CDC25A, RFC4* |
| COLCA∩UCEC | *ANLN, ARHGEF39, ATP8B4, AURKA, CCNE1, CCNF, CDCA2, CDCA5, CDCA8, CENPK, DDX11, DEPDC1B, DSCC1, EME1, ESPL1, FANCI, FEN1, FOXN3, GIMAP6, GTSE1, HMGB3, KNSTRN, MCM2, MCM4, MKI67, MTHFD1L, MTHFD2, NCAPD3, NCAPG, ORC6, PCNA, RAD51AP1, RRM2, SKA1, TIMELESS, TONSL, TPX2, TTK, WDR62, BUB1, CHEK1, LMNB2, PLXNC1, AUNIP, SAPCD2, BLM, DTYMK, EXO1, BUB1B, PRC1, CEP55, FASN, KPNA2, FAT4, SLC9A9, WDHD1, CKAP2L, AURKB, PGAM5, TICRR* |
| PRAD∩BRCA∩COLCA | *-* |
| PRAD∩BRCA∩UCEC | *HJURP, SPAG5, BUB1B, DLGAP5* |
| BRCA∩COLCA∩UCEC | *ANLN, AURKA, CCNE1, CDCA5, DEPDC1B, EME1, SKA1, TTK, CCNF, AUNIP, BUB1, CDCA2, DTYMK, NCAPG, EXO1, KNSTRN* |
| PRAD∩COLCA∩UCEC | *DEPDC1B, BUB1, RRM2* |
| PRAD∩BRCA∩COLCA∩UCEC | *-* |

_PRAD: Prostate adenocarcinoma; BRCA: Breast invasive carcinoma; COLCA: colorectal cancer; UCEC: Uterine Corpus Endometrial Carcinoma._

Supporting Information File 2. List of genes in each GO/KEGG pathway

| **Cancer Combination** | **Terms (Gene Ontology/ Kyoto Encyclopedia of Genes and Genomes pathway)** | **False discovery rate (FDR)** | **Gene lists involved in the given GO/KEGG pathway** |
| --- | --- | --- | --- |
| **PRAD∩BRCA** | mitotic sister chromatid segregation (GO) | 1.44E-19 | *BUB1B, CDCA5, CDCA8, CENPF, DLGAP5, KIF18B, KIF4A, NCAPG, NCAPH, PLK1, PTTG1, SPAG5, TRIP13, TTK, UBE2C* |
|  | mitotic nuclear division (GO) | 1.44E-19 | *BUB1B, CDCA5, CDCA8, CENPF, DLGAP5, KIF18B, KIF4A, MKI67, NCAPG, NCAPH, PKMYT1, PLK1, PTTG1, SPAG5, TRIP13, TTK, UBE2C* |
|  | chromosome segregation (GO) | 7.54E-19 | *BUB1B, CDCA5, CDCA8, CENPF, DLGAP5, HJURP, KIF18B, KIF4A, MKI67, NCAPG, NCAPH, PLK1, PTTG1, SPAG5, TRIP13, TTK, UBE2C* |
|  | organelle fission (GO) | 8.46E-18 | *BUB1B, CDCA5, CDCA8, CENPF, DCN, DLGAP5, KIF18B, KIF4A, MKI67, NCAPG, NCAPH, PKMYT1, PLK1, PTTG1, SPAG5, TRIP13, TTK, UBE2C* |
|  | regulation of chromosome segregation (GO) | 4.41E-15 | *BUB1B, CDCA5, CENPF, DLGAP5, MKI67, PLK1, PTTG1, SPAG5, TRIP13, TTK, UBE2C* |
|  | Cell cycle (KEGG) | 2.26E-05 | *-* |
|  | Oocyte meiosis (KEGG) | 0.008609 | *-* |
| **PRAD∩COLCA** | condensed chromosome (GO) | 0.001566 | *NCAPH, NCAPG, BUB1* |
|  | mitotic chromosome condensation (GO) | 0.002994 | *NCAPH, NCAPG* |
|  | mitotic sister chromatid segregation (GO) | 0.002994 | *NCAPH, NCAPG, BUB1* |
|  | condensed nuclear chromosome (GO) | 0.004676 | *NCAPH, BUB1* |
|  | DNA packaging complex (GO) | 0.004676 | *NCAPH, NCAPG* |
| **PRAD∩UCEC** | chromosome segregation (GO) | 2.38E-18 | *BUB1B, CDCA5, CDCA8, HJURP, MKI67, NEK2, SPAG5, TOP2A, BUB1, AURKB, DLGAP5, NUF2, KIF18B* |
|  | sister chromatid segregation (GO) | 7.40E-17 | *BUB1B, CDCA5, CDCA8, NEK2, SPAG5, TOP2A, BUB1, AURKB, DLGAP5, NUF2, KIF18B* |
|  | nuclear division (GO) | 2.19E-15 | *BUB1B, CDCA5, CDCA8, MKI67, NEK2, SPAG5, TOP2A, BUB1, AURKB, DLGAP5, NUF2, KIF18B* |
|  | mitotic nuclear division (GO) | 2.21E-15 | *BUB1B, CDCA5, CDCA8, MKI67, NEK2, SPAG5, BUB1, AURKB, DLGAP5, NUF2, KIF18B* |
|  | regulation of chromosome segregation (GO) | 4.29E-13 | *BUB1B, CDCA5, MKI67, NEK2, SPAG5, BUB1, AURKB, DLGAP5* |
|  | Cell cycle (KEGG) | 0.042667 | *-* |
| **BRCA∩COLCA** | nuclear division (GO) | 4.61E-20 | *ANLN, AURKA, BUB1, CCNB1, CCNB2, CCNE1, CDCA5, CENPE, CHEK1, EME1, KIF14, KIF2C, KIF4A, KNSTRN, NCAPG, NCAPH, NUSAP1, PSRC1, TTK, MAD2L1, RCC1, FANCA, BUB1B, DLGAP5, RAD54B* |
|  | chromosome segregation (GO) | 5.05E-19 | *BUB1, CCNB1, CCNE1, CDCA2, CDCA5, CENPE, EME1, KIF14, KIF2C, KIF4A, KNSTRN, NCAPG, NCAPH, NUSAP1, PSRC1, SKA1, TTK, MAD2L1, RCC1, BUB1B, SPC25, DLGAP5* |
|  | mitotic nuclear division (GO) | 5.54E-19 | *ANLN, AURKA, BUB1, CCNB1, CCNB2, CDCA5, CENPE, CHEK1, KIF14, KIF2C, KIF4A, KNSTRN, NCAPG, NCAPH, NUSAP1, PSRC1, TTK, MAD2L1, RCC1, BUB1B, DLGAP5* |
|  | mitotic sister chromatid segregation (GO) | 3.59E-16 | *BUB1, CCNB1, CDCA5, CENPE, KIF14, KIF2C, KIF4A, KNSTRN, NCAPG, NCAPH, NUSAP1, PSRC1, TTK, MAD2L1, BUB1B, DLGAP5* |
|  | chromosomal region (GO) | 3.38E-12 | *AURKA, BUB1, CCNB1, CDCA5, CENPE, CHEK1, EZH2, KIF2C, KNSTRN, MC2, NCAPG, RECQL4, SKA1, TTK, MAD2L1, BUB1B, SPC25* |
|  | Cell cycle (KEGG) | 1.97E-12 | *-* |
|  | Oocyte meiosis (KEGG) | 2.05E-05 | *-* |
|  | Progesterone-mediated oocyte maturation (KEGG) | 5.45E-05 | *-* |
|  | p53 signaling pathway (KEGG) | 0.000205 | *-* |
|  | Cellular senescence (KEGG) | 0.00052 | *-* |
| **BRCA∩UCEC** | chromosome segregation (GO) | 2.41E-27 | *BUB1, BUB1B, CCNB1, CCNE1, CDCA5, CDCA8, CENPE, CENPN, DLGAP5, DSC1, EME1, ESCO2, FAM83D, HJURP, KIF18A, KIF4A, KNSTRN, MKI67, NCAPG, PSRC1, RACGAP1, SKA1, SPAG5, TACC3, TTK, ZWINT, SPC25, CDCA2, PRC1, RCC2* |
|  | chromosomal region (GO) | 5.22E-26 | *AURKA, BUB1, BUB1B, CCNB1, CDCA5, CDCA8, CENPE, CENPL, CENPN, CHEK1, DNA2, DSCC1, ESCO2, HJURP, KIF18A, KNSTRN, MCM2, MCM4, NCAPG, ORC1, PIF1, RECQL4, SKA1, SPAG5, TTK, ZWINT, ZWILCH, SPC25, RCC2* |
|  | DNA replication (GO) | 1.50E-22 | *CCNE1, CDC7, CHEK1, DNA2, DSCC1, EME1, ESCO2, GINS1, GINS2, LIG1, MCM10, MCM2, MCM4, ORC1, ORC6, PIF1, POLQ, RECQL4, TICRR, TIMELESS, TONSL, EXO1, RRM2, E2F8, RFC4* |
|  | organelle fission (GO) | 8.05E-22 | *ANLN, AURKA, BUB1, BUB1B, CCNB1, CCNE1, CDCA5, CDCA8, CENPE, CHEK1, DLGAP5, DSCC1, EME1, KIF18A, KIF4A, KNSTRN, MKI67, MND1, MTFR2, NCAPG, PSRC1, RACGAP1, RAD54L, SPAG5, TACC3, TPX2, TTK, ZWINT, PRC1* |
|  | nuclear division (GO) | 8.05E-22 | *ANLN, AURKA, BUB1, BUB1B, CCNB1, CCNE1, CDCA5, CDCA8, CENPE, CHEK1, DLGAP5, DSCC1, EME1, KIF18A, KIF4A, KNSTRN, MKI67, MND1, NCAPG, PSRC1, RACGAP1, RAD54L, SPAG5, TACC3, TPX2, TTK, ZWINT, PRC1* |
|  | Cell cycle (KEGG) | 3.06E-14 | *-* |
|  | DNA replication (KEGG) | 7.94E-06 | *-* |
|  | Mismatch repair (KEGG) | 0.001704 | *-* |
|  | p53 signaling pathway (KEGG) | 0.002887 | *-* |
|  | Cellular senescence (KEGG) | 0.004181 | *-* |
| **COLCA∩UCEC** | chromosome segregation (GO) | 8.31E-18 | *CCNE1, CDCA2, CDCA5, CDCA8, CENPK, DDX11, DSCC1, EME1, ESPL1, FEN1, KNSTRN, MKI67, NCAPD3, NCAPG, SKA1, TTK, BUB1, BUB1B, PRC1, AURKB* |
|  | chromosomal region (GO) | 1.12E-16 | *AURKA, CDCA5, CDCA8, CENPK, DSCC1, FEN1, KNSTRN, MCM2, MCM4, NCAPD3, NCAPG, PCNA, SKA1, TTK, BUB1, CHEK1, BLM, BUB1B, AURKB* |
|  | mitotic nuclear division (GO) | 2.86E-16 | *ANLN, AURKA, CDCA5, CDCA8, CENPK, DSCC1, ESPL1, KNSTRN, MKI67, NCAPD3, NCAPG, TPX2, TTK, BUB1, CHEK1, BUB1B, PRC1, AURKB* |
|  | nuclear division (GO) | 5.24E-16 | *ANLN, AURKA, CCNE1, CDCA5, CDCA8, CENPK, DSCC1, EME1, ESPL1, KNSTRN, MKI67, NCAPD3, NCAPG, TPX2, TTK, BUB1, CHEK1, BUB1B, PRC1, AURKB* |
|  | DNA replication (GO) | 1.42E-15 | *CCNE1, DDX11, DSCC1, EME1, FEN1, MCM2, MCM4, ORC6, PCNA, RRM2, TIMELESS, TONSL, CHEK1, BLM, EXO1, WDHD1, TICRR* |
|  | Cell cycle (KEGG) | 2.88E-10 | *-* |
|  | DNA replication (KEGG) | 0.000148 | *-* |
|  | p53 signaling pathway (KEGG) | 0.001661 | *-* |
|  | Fanconi anemia pathway (KEGG) | 0.007581 | *-* |
|  | Oocyte meiosis (KEGG) | 0.007581 | *-* |

_PRAD: Prostate adenocarcinoma; BRCA: Breast invasive carcinoma; COLCA: colorectal cancer; UCEC: Uterine Corpus Endometrial Carcinoma._
